# Circular RNA MCTP2 inhibits cisplatin resistance in gastric cancer by miR-99a-5p-mediated induction of MTMR3 expression

**DOI:** 10.1186/s13046-020-01758-w

**Published:** 2020-11-17

**Authors:** Guangli Sun, Zheng Li, Zhongyuan He, Weizhi Wang, Sen Wang, Xing Zhang, Jiacheng Cao, Penghui Xu, Haixiao Wang, Xiaoxu Huang, Yiwen Xia, Jialun Lv, Zhe Xuan, Tianlu Jiang, Lang Fang, Jing Yang, Diancai Zhang, Hao Xu, Zekuan Xu

**Affiliations:** 1 Department of General Surgery, The First Affiliated Hospital of Nanjing Medical University, Nanjing, Jiangsu province China; 2 Collaborative Innovation Center for Cancer Personalized Medicine, Nanjing Medical University, Nanjing, Jiangsu province China

**Keywords:** GC, Cisplatin, CircMCTP2, miR-99a-5p, MTMR3

## Abstract

**Background:**

Cisplatin (CDDP) is the first-line chemotherapy for gastric cancer (GC). The poor prognosis of GC patients is partially due to the development of CDDP resistance. Circular RNAs (circRNAs) are a subclass of noncoding RNAs that function as microRNA (miRNA) sponges. The role of circRNAs in CDDP resistance in GC has not been evaluated.

**Methods:**

RNA sequencing was used to identify the differentially expressed circRNAs between CDDP-resistant and CDDP-sensitive GC cells. qRT-PCR was used to detect the expression of circMCTP2 in GC tissues. The effects of circMCTP2 on CDDP resistance were investigated in vitro and in vivo. Pull-down assays and luciferase reporter assays were performed to confirm the interactions among circMCTP2, miR-99a-5p, and myotubularin-related protein 3 (MTMR3). The protein expression levels of MTMR3 were detected by western blotting. Autophagy was evaluated by confocal microscopy and transmission electron microscopy (TEM).

**Results:**

CircMCTP2 was downregulated in CDDP-resistant GC cells and tissues compared to CDDP-sensitive GC cells and tissues. A high level of circMCTP2 was found to be a favorable factor for the prognosis of patients with GC. CircMCTP2 inhibited proliferation while promoting apoptosis of CDDP-resistant GC cells in response to CDDP treatment. CircMCTP2 was also found to reduce autophagy in CDDP-resistant GC cells. MiR-99a-5p was verified to be sponged by circMCTP2. Inhibition of miR-99a-5p could sensitize GC cells to CDDP. MTMR3 was confirmed to be a direct target of miR-99a-5p. Knockdown of MTMR3 reversed the effects of circMCTP2 on the proliferation, apoptosis and autophagy of CDDP-resistant GC cells. CircMCTP2 was also confirmed to inhibit CDDP resistance in vivo in a nude mouse xenograft model.

**Conclusions:**

CircMCTP2 sensitizes GC to CDDP through the upregulation of MTMR3 by sponging miR-99a-5p. Overexpression of CircMCTP2 could be a new therapeutic strategy for counteracting CDDP resistance in GC.

**Supplementary Information:**

The online version contains supplementary material available at 10.1186/s13046-020-01758-w.

## Background

GC is one of the most common malignancies in the world and is the fourth and fifth most prevalent malignancy in men and women, respectively [1]. Compared with other areas of the world, GC is more common in East Asia, with 43% of GC patients in China alone [2]. Despite improved treatment for GC, the prognosis of advanced GC patients remains poor, with a low 5-year survival rate [3]. CDDP-based chemotherapy is the main treatment strategy for patients with advanced GC [4]. However, after several cycles of chemotherapy, 50% of the patients exhibit acquired drug resistance. The 5-year survival rate of these CDDP-resistant patients is only approximately 20% [5–7]. Thus, it is of great importance to elucidate the underlying molecular mechanisms involved in CDDP resistance in GC.

CircRNAs are endogenous noncoding RNAs that are characterized by closed loops [8]. It has been shown that circRNAs are produced from the back-splicing of pre-mRNA, and they are conserved and stable because of their special structure [9, 10]. Recently, circRNAs have garnered attention, as they have been found to act as miRNA sponges [11]. MiRNAs regulate gene expression by binding to the 3′-untranslated regions (3′-UTRs) of target genes [12]. Thus, through their action on miRNAs, circRNAs can regulate the expression and biological functions of their target genes. CircRNAs play crucial roles in many types of carcinomas. CircNRIP1 promotes GC progression by sponging miR-149-5p [13]. CircMTO1 can inhibit the progression of hepatocellular carcinoma through the regulation of miR-9-mediated expression of p21 [14]. CircTP63 has been shown to contribute to the progression of lung squamous cell carcinoma by acting as a sponge of miR-873–3p [15]. However, the relationship between circRNAs and CDDP resistance in GC remains unknown.

It has been reported that miR-99a-5p is upregulated in CDDP-resistant GC cell lines compared to corresponding sensitive cell lines and that miR-99a-5p promotes GC cell resistance to CDDP [16]. MiR-99a-5p has also been verified to be negatively correlated with the prognosis of patients with GC [17].

Autophagy is an intracellular system that removes damaged and unnecessary cellular components, such as damaged organelles and proteins, by delivering them to lysosomes for degradation [18]. Accumulative studies have shown that autophagy is involved in CDDP resistance in different kinds of carcinomas [19, 20]. Inhibition of autophagy has also been reported to sensitize GC cells to CDDP [21, 22]. MTMR3 is one of the members of the myotubularin family. It is an inositol lipid 3-phosphatase that can hydrolyze PtdIns3P (PI3P) [23]. PI3P is required for the process of autophagy [24]. The suppressive effect of MTMR3 on PI3P was found to inhibit autophagosome formation [25]. Nevertheless, whether MTMR3 is involved in the regulation of CDDP resistance in GC cells has not been elucidated.

In this study, circMCTP2 was detected by RNA sequencing as one of the circular RNAs that was downregulated in CDDP-resistant GC cells compared to CDDP-sensitive GC cells. We report that circMCTP2 acts as a miR-99a-5p sponge and sensitizes GC cells to CDDP through the upregulation of MTMR3. We believe that our findings may be helpful for the treatment of patients with CDDP-resistant GC.

## Methods

### Tissue samples

All GC tissues used in this study were obtained from the First Affiliated Hospital of Nanjing Medical University. All GC patients received CDDP-based chemotherapy after radical gastrectomy. Tumor marker evaluation was performed after each cycle of chemotherapy. Abdominal contrast-enhanced CT was performed after every two cycles of chemotherapy. Based on the results of tumor marker examination and imaging examination, we could judge whether the tumor recurred. CDDP resistance was defined as tumor recurrence during CDDP-based chemotherapy after radical gastrectomy, and CDDP sensitivity was defined as no tumor relapse during CDDP-based therapy [26]. The tissues used for RNA extraction and immunochemical staining were stored in liquid nitrogen and 4% formaldehyde, respectively.

### Cell lines

The BGC823, SGC7901, and SGC7901CDDP cell lines were purchased from the Cell Bank of Type Culture Collection of Chinese Academy of Sciences. The BGC823CDDP cell line was established according to a published protocol [27]. The four cell lines were cultured in RPMI 1640 (Gibco, USA) at 37 °C with 5% carbon dioxide in an incubator.

### RNA extraction and RNase R treatment

The Cytoplasmic and Nuclear RNA Purification Kit (Norgen Biotek, Canada) was used to extract nuclear and cytoplasmic RNAs. Total RNA from GC tissues and cells was extracted using TRIzol reagent (Invitrogen, USA). The extracted total RNA of GC cell lines was mixed with 3 U/mg RNase R for 15 min at 37 °C. qRT-PCR was performed to detect the expression of circMCTP2 and MCTP2 mRNA, which indicated the stability of circMCTP2 and MCTP2 mRNA.

### Quantitative real-time polymerase chain reaction

mRNA was reverse transcribed into cDNA using the Prime script RT Reagent (Takara, Japan). For reverse transcription of miRNA, we used a New Poly(A) Tailing Kit (ThermoFisher Scientific, China). qRT-PCR was carried out with an ABI StepOne Plus system using SYBR Green Master Mix (Roche, USA). The primers are listed in Additional file 1: Table S1.

### Actinomycin D assay

GC cells (5 × 10^4^ cells per well) were seeded into a 24-well plate. After 24 h, GC cells were mixed with 2 mg/L actinomycin D (Sigma-Aldrich, USA) for 0, 4, 8, 12, and 24 h. The stabilities of circRNA and mRNA were examined by qRT-PCR.

### Fluorescence in situ hybridization (FISH)

We followed a previously published protocol to perform the assay [28]. We used a biotin-labeled probe for circMCTP2 and a Dig-labeled probe for miR-99a-5p (Exiqon, Denmark) in this assay. The signals of the biotin-labeled probe and the Dig-labeled probe were captured using Cy5-conjugated streptavidin and a tyramide-conjugated Alexa 488 fluorochrome TSA kit (Thermo Fisher Scientific, China), respectively. Nuclei were stained with DAPI.

### Cell transfection

Commercially available lentivirus-circMCTP2, lentivirus-miR-99a-5p mimics, lentivirus-miR-99a-5p inhibitor, and lentivirus-shMTMR3 were purchased from GenePharma (Shanghai, China). After transfection, we used puromycin (Solarbio, China) to establish stably transfected cell lines.

### Cell counting kit-8 (CCK-8) cell viability assay

CCK-8 (Dojindo, Japan) was used to determine the cell viability of GC cells. GC cells were seeded into a 96-well plate at a density of 5000 cells per well. Seeded cells were incubated for 2 h with the CCK-8 reagent before measurement.

### EdU assay

We performed the EdU assay to assess DNA synthesis, which indicated GC cell proliferation, using an EdU assay kit (RiboBio, China). Stained GC cells were photographed using a microscope (Nikon, Japan).

### Flow cytometric analysis

GC cells were seeded at a density of 2 × 10^5^ cells per well in a 6-well plate. After treatment with CDDP for 48 h, a PI/Annexin V Apoptosis Detection Kit (BD, USA) was used to stain the collected GC cells. The proportion of apoptotic GC cells was detected using a flow cytometer (Gallios, Beckman, USA).

### Colony formation assay

GC cells were seeded into a 6-well plate at a density of 1000 cells per well. After being cultured for 14 days, crystal violet (Kaigen, China) was used to stain the fixed GC cells. The cells were washed with PBS, and then, the number of colonies was counted.

### Luciferase reporter assay

Wild-type (wt) and mutant (mut) sequences of circMCTP2 were designed and inserted into the pGL-3 luciferase reporter vector (Realgene, China). BGC823CDDP and SGC7901CDDP were cotransfected with luciferase reporter plasmids and miR-99a-5p mimics. Firefly and Renilla luciferase activities were determined using the Dual-Luciferase Reporter Assay System (Promega, USA).

### RNA pull-down assay

The RNA pull-down assay was performed per previously described methods [29]. RiboBio (Guangzhou, China) designed and synthesized a biotin-labeled probe specific to circMCTP2. GC cells were collected and sonicated to produce cell lysates. The lysate was incubated with circMCTP2 probe that was prebound to streptavidin-coupled Dynabeads (Invitrogen, USA) and oligo probe. The RNA mixture bound to the magnetic beads was rinsed with wash buffer and then extracted using the RNeasy Mini Kit (QIAGEN, Germany). We performed qRT-PCR on the RNA product. Biotinylated-miR-99a-5p and biotinylated-miR-NC were produced by GenePharma (Shanghai, China). After 48 h, the constructs were transfected into GC cells, and the cells were lysed. The lysate was incubated with streptavidin-coated magnetic beads and then rinsed with PBS. The biotin-coupled RNA complex was pulled down and subjected to qRT-PCR.

### RNA immunoprecipitation assay

A Magna RNA immunoprecipitation (RIP) kit (Millipore, USA) was used to perform the RIP assay. We used RIP buffer to lyse GC cells, and the cell lysate was then incubated with magnetic beads conjugated with anti-Ago2 antibody (Millipore, USA) or IgG antibody. Finally, the immunoprecipitated RNA was extracted and subjected to qRT-PCR.

### Transmission electron microscopy (TEM)

Prepared GC cells were harvested, and a 2.5% solution of glutaraldehyde was used to fix GC cells overnight. Then, the GC cells were fixed with 1% OSO4 for 1 h. Samples were dehydrated with increasing concentrations of ethanol, followed by embedding in Epon. Sections were cut with an ultramicrotome and then stained with 0.3% lead citrate. A JEM-1010 electron microscope (JEOL, Japan) was used to observe autophagy in GC cells.

### Confocal microscopy

GC cells transfected with GFP-mRFP-LC3 lentivirus (GeneChem, China) were seeded into a 35-mm culture dish for confocal microscopy. Hoechst was used to stain nuclei. Red and yellow puncta representing autolysosomes and autophagosomes, respectively, were detected by confocal microscopy (Carl Zeiss, Germany). Three random fields were selected for the quantification of puncta.

### Western blotting

Total protein was extracted from GC cells and tissues. The proteins were transferred to PVDF membranes (Millipore USA) after SDS-PAGE electrophoresis. After blocking in TBST buffer with 5% skimmed milk, the membranes were incubated with primary antibodies overnight at 4 °C. The membranes were rinsed three times with TBST and then incubated with secondary antibodies at room temperature for 2 h. After the membranes were washed with TBST three times, the bands were detected using an enhanced chemiluminescence detection system with Chemiluminescence HRP Substrate (Millipore, WBKL0100). Anti-Bcl2, anti-Bax, anti-caspase3, anti-c-caspase3, anti-β-actin, anti-P62, anti-Beclin1, anti-LC3, and anti-MTMR3 were purchased from Abcam (Cambridge, UK). Anti-rabbit IgG-HRP and anti-mouse IgG-HPR antibodies were obtained from Santa Cruz (Dallas, TX, USA).

### Nude mouse xenograft model

Female BALB/c nude mice (5 weeks old) were purchased from the Department of Laboratory Animal Center of Nanjing Medical University. Stably transfected GC cells (1 × 10^6^) were injected subcutaneously into each armpit of a nude mouse. A week later, CDDP (5 mg/kg) was injected intraperitoneally into nude mice three times per week. All nude mice were sacrificed at day 35.

### TUNEL assay

We performed TUNEL assays to measure the rate of GC cell apoptosis rate in nude mouse subcutaneous tumors. The assay was carried out using a Cell Death Detection Kit (Roche, USA), and TUNEL-positive cells were counted using a microscope (Nikon, Japan).

### Immunochemical staining

Tissue samples from GC patients and subcutaneous tumors from nude mice were fixed with 4% formalin and embedded in paraffin. The sections were incubated with anti-ki67, anti-P62, and anti-MTMR3 antibodies overnight at 4 °C. Then, the sections were incubated with secondary antibody for 1 h at room temperature and developed with DAB solution for 3 min. The sections were counterstained with hematoxylin.

### Statistical analysis

Statistical Product and Service Solutions (SPSS) software version 19.0 was used for statistical analysis. All experiments were performed in triplicate, and the results are expressed as the mean value ± standard deviation (SD). Student’s t-test was used to determine significant differences between two independent groups. Pearson’s *χ*^*2*^ test was used to analyze the relationship between the circMCTP2 expression level and clinicopathological features of the GC patients. The survival analysis was performed with the Kaplan-Meier method. The differences in survival between the two groups were assessed using the log-rank test. Linear correlation analysis was performed to determine the correlations between gene expression levels. *P* < 0.05 (*) or *P* < 0.01 (**) was considered statistically significant.

## Results

### CircMCTP2 is downregulated in CDDP-resistant GC cells and tissues

**Fig. 1:**
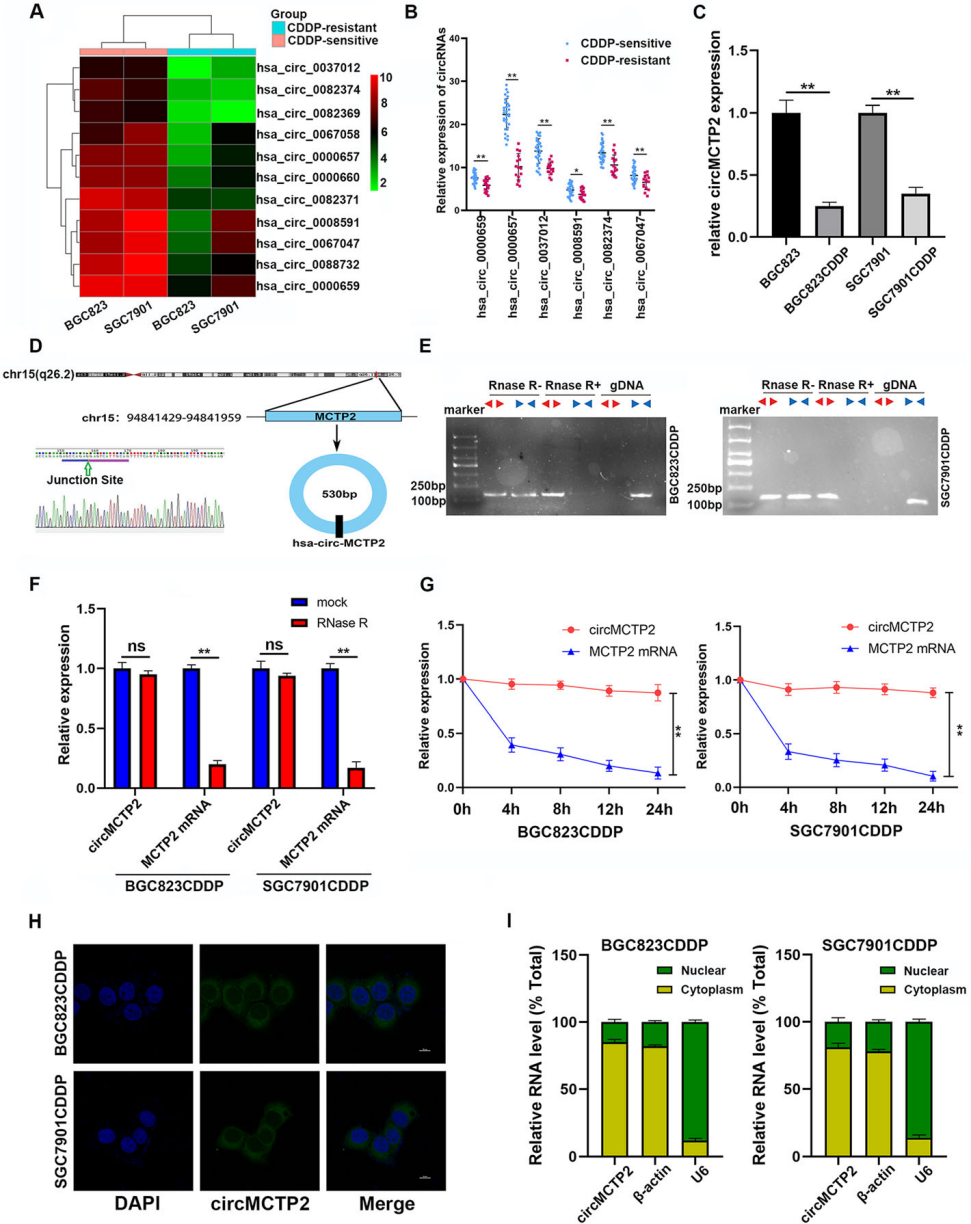
CircMCTP2 expression is downregulated in CDDP-resistant GC tissues and cells. **a** Heatmap of circular RNAs downregulated in BGC823CDDP and SGC7901CDDP cells compared to CDDP-sensitive GC cells. **b** qRT-PCR-based identification of circular RNAs that were decreased in CDDP-resistant GC tissues. **c** Expression of circMCTP2 in CDDP-resistant and CDDP-sensitive GC cells by qRT-PCR. **d** Confirmation of head-to-tail splicing of circMCTP2 with Sanger sequencing. **e** RT-PCR-based detection of back-spliced and linear MCTP2 in cDNA with or without RNase R and genomic DNA (gDNA). **f** CircMCTP2 and MCTP2 mRNA were treated with RNase R to detect their stability. **g** Results of actinomycin D assay. **h** FISH-based validation of preferential localization of circMCTP2 to the GC cell cytoplasm. **i** qRT-PCR-based confirmation of circMCTP2 localization mainly within the GC cell cytoplasm. (*p < 0.05, **p < 0.01. Data are expressed as the means ± SDs)

To generate the profile of differentially expressed circular RNAs in CDDP-sensitive and CDDP-resistant GC cells, we performed RNA-seq analysis of BGC823 (CDDP-sensitive), BGC823CDDP (CDDP-resistant), SGC7901 (CDDP-sensitive), and SGC7901CDDP (CDDP-resistant) cells. Hundreds of circular RNAs were found to be downregulated in CDDP-resistant GC cells compared to CDDP-sensitive GC cells. We selected the top 50 downregulated circular RNAs each in BGC823CDDP and SGC7901CDDP cells and found that 11 circular RNAs were downregulated in both BGC823CDDP and SGC7901CDDP cells (Fig. 1a). To further verify whether these circular RNAs were reduced in the CDDP-resistant GC tissues, qRT-PCR was performed on 45 CDDP-sensitive and 15 CDDP-resistant GC tissues. Only six of them were decreased in the CDDP-resistant GC tissues, and circMCTP2 (hsa_circ_0000657) was found to be the most downregulated circRNA (Fig. 1b). CircMCTP2 was also determined to be reduced in CDDP-resistant GC cells by qRT-PCR (Fig. 1c). We expanded the sample size of GC tissues to 75 CDDP-sensitive and 25 CDDP-resistant GC tissues and then examined the expression levels of circMCTP2. CircMCTP2 was found to be downregulated in CDDP-resistant GC tissues with the larger sample size (Additional file 2: Fig. S1a). The ROC curve was drawn according to the expression of circMCTP2 in the GC tissues (Additional file 2: Fig. S1b). The area under the curve (AUC) for distinguishing CDDP-resistant from CDDP-sensitive patients was 0.9450, which implied that circMCTP2 could be a predictive biomarker for CDDP resistance in GC. The patients with GC were divided into two groups based on the expression levels of circMCTP2, with 50 patients in each group. Based on the survival information we had collected earlier, DFS and OS curves were drawn. The patients with high expression of circMCTP2 showed a better prognosis than those in the low expression group (Additional file 2: Fig. S1c and S1d). Analysis of the clinicopathological characteristics showed that the expression of circMCTP2 was negatively correlated with tumor progression and CDDP resistance (Table 1).

**Table 1:** Expression of circMCTP2 in human gastric cancer and the clinicopathological characteristics of the patients’

Characteristics	Number	circMCTP2 expression		*P*-value
		High group	Low group	
Age (years)				
≥ 60	61	32	29	0.539
< 60	39	18	21	
Gender				
Male	68	32	36	0.391
Female	32	18	14	
Size (cm)				
≥ 3(cm)	57	21	36	0.002**
< 3(cm)	43	29	14	
Stage				
II	42	26	16	0.043*
III	58	24	34	
T grade				
T1 + T2	36	23	13	0.037*
T3 + T4	64	27	37	
Lymph node metastasis				
N1-N3	87	41	46	0.137
N0	13	9	4	
CDDP chemosensitivity				
Sensitive	75	48	27	< 0.001**
Resistant	25	2	3	

*p < 0.05 and **p < 0.01 Statistically significant difference

### Identification of circMCTP2 in GC

CircMCTP2 is derived from the MCTP2 locus that is located on chromosome 15. The head-to-tail structure of circMCTP2 was identified by Sanger sequencing (Fig. 1d). Convergent and divergent primers were designed for the amplification of linear MCTP2 and circMCTP2, respectively. As shown in Fig. 1e, circMCTP2 could be detected in cDNA under RNase R treatment, whereas linear MCTP2 was digested with RNase R. Linear MCTP2, rather than circMCTP2, could be detected in gDNA. Under RNase R treatment, circMCTP2 showed more stability than MCTP2 mRNA (Fig. 1f). GC cells were treated with actinomycin D to further examine the stability of circMCTP2. As shown in Fig. 1g, circMCTP2 was more stable than MCTP2 mRNA. The FISH assay results revealed that circMCTP2 was predominantly located in the GC cell cytoplasm (Fig. 1h). CircMCTP2 was also confirmed by qRT-PCR to be localized mainly within the cytoplasm (Fig. 1i). These results suggest that circMCTP2 is a stable cytoplasmic circRNA in GC cells.

### CircMCTP2 inhibits CDDP resistance in GC in vitro

**Fig. 2:**
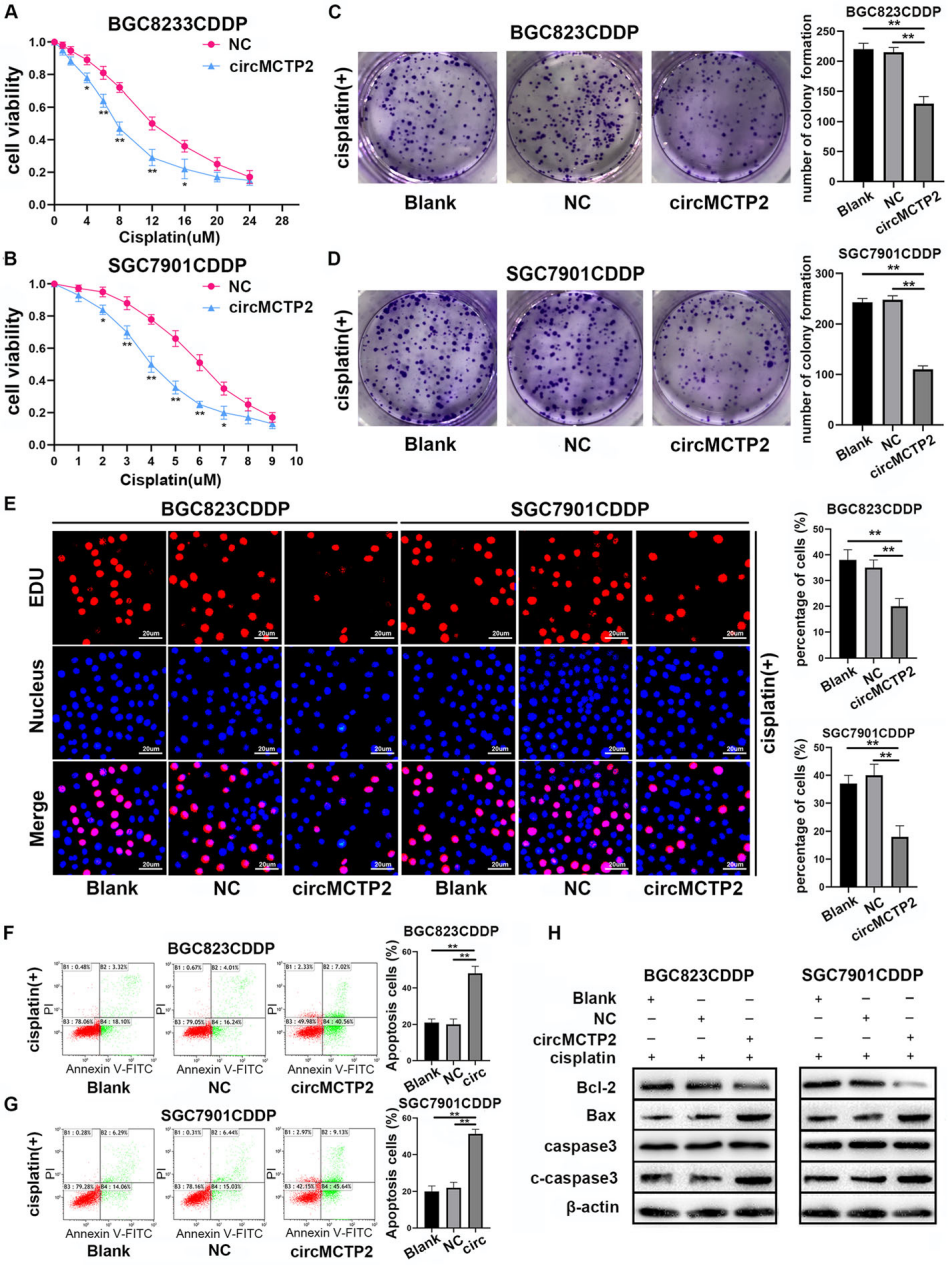
Overexpression of circMCTP2 facilitates the sensitivity of CDDP-resistant GC cells in vitro. **a** and **b** Reduction in CDDP-resistant GC cell viability by elevated expression of circMCTP2. **c** Impairment of the colony-forming ability of BGC823CDDP cells in response to cisplatin treatment after circMCTP2 overexpression. **d** CircMCTP2-mediated decrease in colony formation by SGC7901CDDP cells in the presence of cisplatin. **e** Assessment of DNA synthesis of BGC823CDDP and SGC7901CDDP cells transfected with NC and lentivirus-circMCTP2 in the presence of cisplatin. **f** and **g** Flow cytometric analysis of the apoptosis of CDDP-resistant GC cells treated with cisplatin. **h** Detection of apoptosis-related proteins by western blotting in BGC823CDDP and SGC7901CDDP cells. CDDP treatment: 12 μM for 48 h in BGC823CDDP cells and 6 μM for 48 h in SGC7901CDDP cells. (*p < 0.05, **p < 0.01. Data are expressed as the means ± SDs)

Because circMCTP2 was downregulated in CDDP-resistant GC cells, we transfected the BGC823CDDP and SGC7901CDDP cell lines with lentivirus-circMCTP2. We observed that circMCTP2 was overexpressed in both cell lines after lentivirus transfection, whereas the level of MCTP2 mRNA was not affected (Additional file 3: Fig. S2a and S2b). Overexpression of circMCTP2 reduced BGC823CDDP and SGC7901CDDP viability in response to treatment with different concentrations of CDDP (Fig. 2a and b). Elevated levels of circMCTP2 decreased the number of colonies formed by BGC823CDDP and SGC7901CDDP cells (Fig. 2c and d). As shown in Fig. 2e, CDDP-resistant GC cell proliferation was impaired by the overexpression of circMCTP2. Increased apoptosis induction in response to upregulation of circMCTP2 was detected in BGC823CDDP and SGC7901CDDP cells by flow cytometric analysis (Fig. 2f and g). The results of western blotting also showed that circMCTP2 could promote apoptosis of CDDP-resistant GC cells in response to CDDP treatment (Fig. 2h).

### CircMCTP2 represses autophagy in CDDP-resistant GC cells

**Fig. 3:**
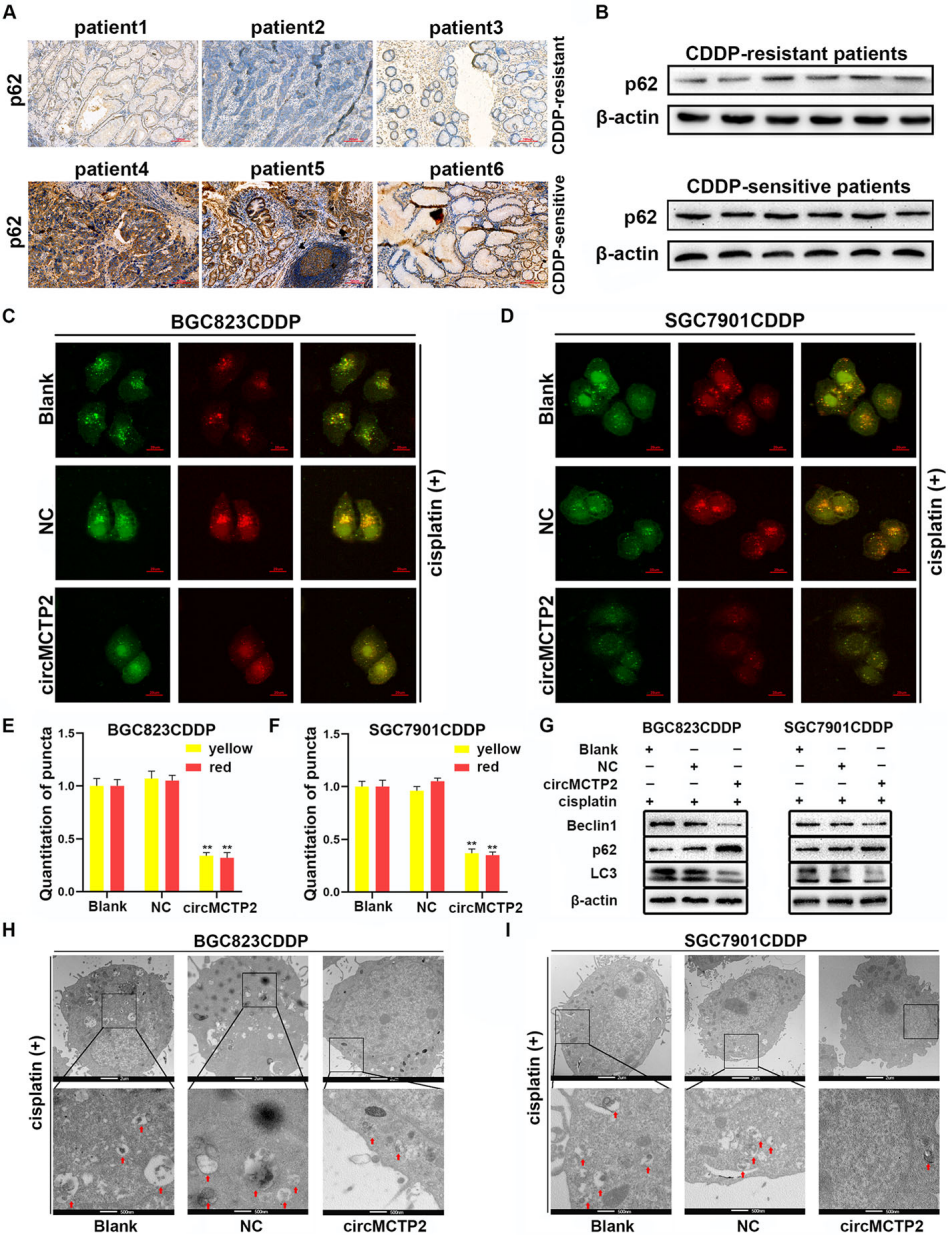
CircMCTP2 represses autophagy in CDDP-resistant GC cells. **a** Expression of p62 protein was lower in CDDP-resistant GC patients than in CDDP-sensitive GC patients. **b** Expression levels of p62 were detected in GC tissues by western blotting. **c-f** GFP/mRFP-LC3 dots were observed and counted by confocal microscopy. **g** Protein levels of Beclin1, p62, and LC3-II were examined by western blotting. **h** and **i** The autophagic microstructure of CDDP-resistant GC cells was observed by transmission electron microscopy. CDDP treatment: 12 μM for 48 h in BGC823CDDP cells and 6 μM for 48 h in SGC7901CDDP cells. (*p < 0.05, **p < 0.01. Data are expressed as the means ± SDs)

Enhancement of autophagy has been reported to contribute to CDDP resistance [30, 31]. We detected p62 by immunohistochemistry (IHC) and western blotting in CDDP-resistant and CDDP-sensitive GC tissues. As shown in Fig. 3a and b, p62 expression was lower in the CDDP-resistant patients, suggesting that autophagy might play a promotive role in CDDP resistance. Then, we investigated the effect of circMCTP2 on autophagy. We observed that the accumulation of GFP/mRFP-LC3 dots was repressed by the overexpression of circMCTP2 (Fig. 3c-f). The expression of LC3-II protein was observed to be decreased by the overexpression of circMCTP2 (Fig. 3g). The results of transmission electron microscopy (TEM) also revealed that circMCTP2 could inhibit autophagy in CDDP-resistant GC cells (Fig. 3h and i).

### CircMCTP2 acts as a sponge of miR-99a-5p

**Fig. 4:**
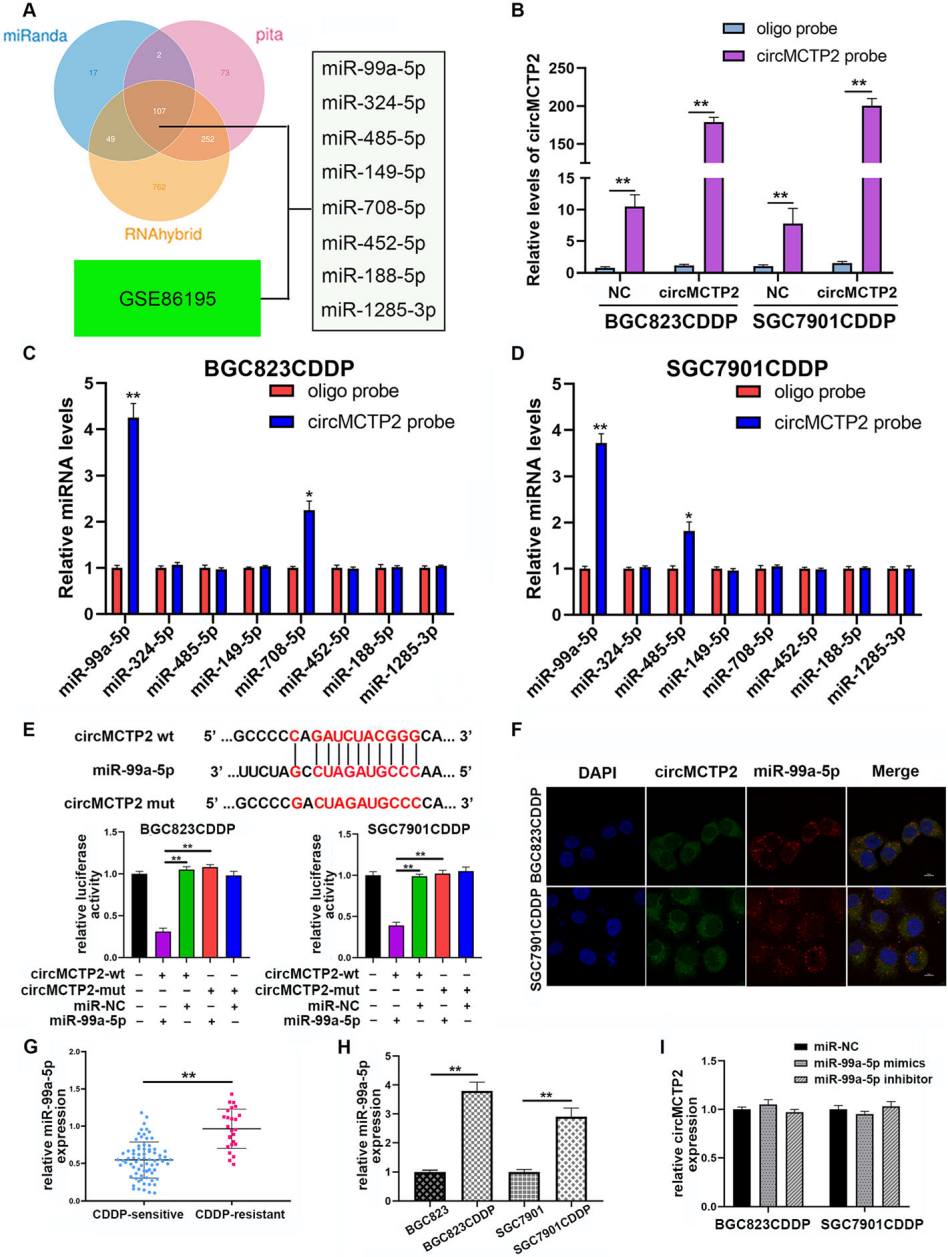
CircMCTP2 serves as a miRNA sponge of miR-99a-5p. **a** Candidate miRNAs predicted to be the potential binding targets of circMCTP2. **b** qRT-PCR analysis to examine the expression of circMCTP2 in BGC823CDDP and SGC7901CDDP cell lysates. **c** and **d** qRT-PCR analysis to examine the expression of candidate miRNAs after pull-down assays. **e** Analysis of luciferase activity in BGC823CDDP and SGC7901CDDP cells transfected with miR-NC or miR-99a-5p mimics and WT or mutant circMCTP2. **f** CircMCTP2 and miR-99a-5p localization to the cytoplasm of CDDP-resistant GC cells by FISH assay. **g** MiR-99a-5p upregulation in CDDP-resistant GC tissues by qRT-PCR. **h** MiR-99a-5p upregulation in CDDP-resistant GC cells by qRT-PCR. **i** Lack of effect of miR-99a-5p overexpression or knockdown on the expression of circMCTP2. (*p < 0.05, **p < 0.01. Data are expressed as the means ± SDs)

Three online databases, namely, miRanda, PITA, and RNAhybrid, were used to predict the potential miRNAs that could be sponged by circMCTP2. The results of a microarray, which was conducted to detect miRNAs differentially expressed in CDDP-sensitive and CDDP-resistant GC cells, were downloaded from the GEO database (GSE86195). Eight miRNAs (miR-99a-5p, miR-324-5p, miR-485-5p, miR-149-5p, miR-708-5p, miR-452-5p, miR-188-5p, and miR-1285-3p) were predicted to be potential targets of circMCTP2 and were upregulated in CDDP-resistant GC cells (Fig. 4a). We then performed the RNA pull-down assay using a biotin-labeled circMCTP2 probe. As shown in Fig. 4b, the pull-down efficiency was enhanced by the overexpression of circMCTP2. The expression levels of the candidate miRNAs were examined by qRT-PCR after pull-down. MiR-99a-5p was pulled down by a biotin-labeled circMCTP2 probe in BGC823CDDP and SGC7901CDDP cells (Fig. 4c and d). The results of the luciferase assay showed that the overexpression of miR-99a-5p could decrease the luciferase activity of the WT circMCTP2 reporter rather than the mutated circMCTP2 reporter, which indicated that miR-99a-5p could directly bind to circMCTP2 (Fig. 4e). The FISH assay results revealed that circMCTP2 and miR-99a-5p were both localized to the BGC823CDDP and SGC7901CDDP cell cytoplasm (Fig. 4f). By qRT-PCR, we found that miR-99a-5p was upregulated in CDDP-resistant GC tissues (Fig. 4g). MiR-99a-5p levels were also found to be enhanced in CDDP-resistant GC cells (Fig. 4h). Neither overexpression nor knockdown of miR-99a-5p had any influence on the expression levels of circMCTP2 (Fig. 4i). There were no significant changes in miR-99a-5p expression after overexpressing circMCTP2 in CDDP-resistant GC cells (Additional file 4: Fig. S3a). In addition, there was no linear correlation between the expression levels of circMCTP2 and miR-99a-5p in CDDP-resistant GC tissues (Additional file 4: Fig. S3b). These results suggested that miR-99a-5p was sponged rather than digested by circMCTP2.

### Knockdown of miR-99a-5p inhibits CDDP resistance in GC cells

Considering that miR-99a-5p is upregulated in BGC823CDDP and SGC7901CDDP cells, we transfected BGC823CDDP and SGC7901CDDP cells with lentivirus-miR-99a-5p inhibitor. The transfection efficiency was examined by qRT-PCR (Additional file 3: Fig. S2c and S2d). Downregulation of miR-99a-5p repressed the proliferation of BGC823CDDP and SGC7901CDDP cells (Additional file 5: Fig. S4a and S4b). Then, we performed flow cytometric analysis and western blotting to explore the influence of miR-99a-5p on cell apoptosis. We observed that apoptosis of CDDP-resistant GC cells was enhanced by the knockdown of miR-99a-5p (Additional file 5: Fig. S4c and S4d).

### MTMR3 is a direct target of miR-99a-5p

**Fig. 5:**
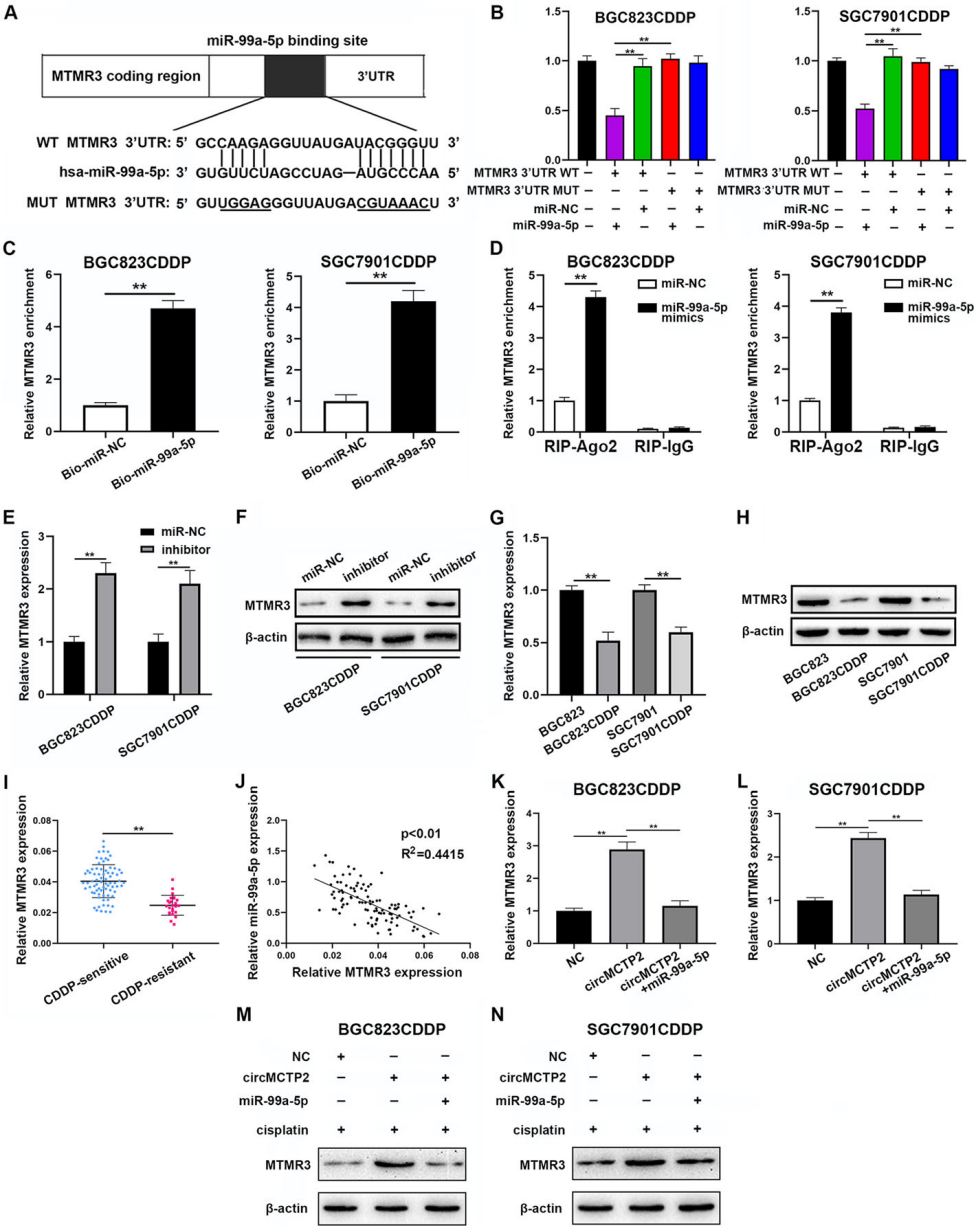
MTMR3 is a direct target of miR-99a-5p. **a** Potential binding site of miR-99a-5p for MTMR3 as predicted by TargetScan. **b** Luciferase reporter assay for determining whether miR-99a-5p could bind to wt-MTMR3–3’UTR or mut-MTMR3–3’UTR. **c** Pull-down assay confirmation of MTMR3 as a target of miR-99a-5p. **d** RIP assay confirmation of the interaction between miR-99a-5p and MTMR3. **e** qRT-PCR analysis revealing that downregulation of miR-99a-5p could increase the expression levels of MTMR3. **f** Increased protein expression levels of MTMR3 after knockdown of miR-99a-5p. **g** qRT-PCR analysis of the expression of MTMR3 CDDP-sensitive and CDDP-resistant GC cells. **h** MTMR3 detection in CDDP-resistant and CDDP-sensitive GC cells by western blotting. **i** MTMR3 expression detection using qRT-PCR in CDDP-sensitive and CDDP-resistant GC tissues. **j** Negative correlation between MTMR3 expression and miR-99a-5p expression. **k** and **l** qRT-PCR analysis showing that MTMR3 expression was increased by circMCTP2 but reversed by overexpression of miR-99a-5p. **m** and **n** Diminishment of the promotive effect of circMCTP2 on the protein expression of MTMR3 by the overexpression of miR-99a-5p. (*p < 0.05, **p < 0.01. Data are expressed as the means ± SDs)

MTMR3 was predicted to be a potential target of miR-99a-5p by four online databases (miRDB, miRWalk, TargetScan and miRTarBase) (Fig. 5a). Additionally, previous studies have confirmed that MTMR3 can inhibit autophagy [25]. Therefore, we hypothesized that MTMR3 might be a direct target of miR-99a-5p. Then, we performed a luciferase reporter assay to confirm the binding of MTMR3 to miR-99a-5p in BGC823CDDP and SGC7901CDDP cells (Fig. 5b). The results of the pull-down assay also confirmed the interaction between miR-99a-5p and MTMR3 (Fig. 5c). Additionally, as shown in Fig. 5d, overexpressed miR-99a-5p caused MTMR3 enrichment in BGC823CDDP and SGC7901CDDP cells after Ago2 RIP. Knockdown of miR-99a-5p increased the expression levels of MTMR3 (Fig. 5e). Western blotting also confirmed that the protein expression levels of MTMR3 were negatively correlated with the expression of miR-99a-5p (Fig. 5f). MTMR3 was observed to be downregulated in CDDP-resistant GC cells (Fig. 5g and h). MTMR3 expression was also found to be reduced in CDDP-resistant GC tissues by qRT-PCR (Fig. 5i). As shown in Fig. 5j, there was a negative correlation between the expression levels of miR-99a-5p and MTMR3. As shown in Fig. 5k-n, MTMR3 expression was increased by circMCTP2, and this effect could be reversed by the overexpression of miR-99a-5p. We confirmed that MTMR3 was directly targeted by miR-99a-5p and that circMCTP2 could upregulate MTMR3 by sponging miR-99a-5p.

### Knockdown of MTMR3 reverses the effect of circMCTP2 on CDDP-resistant GC cells

**Fig. 6:**
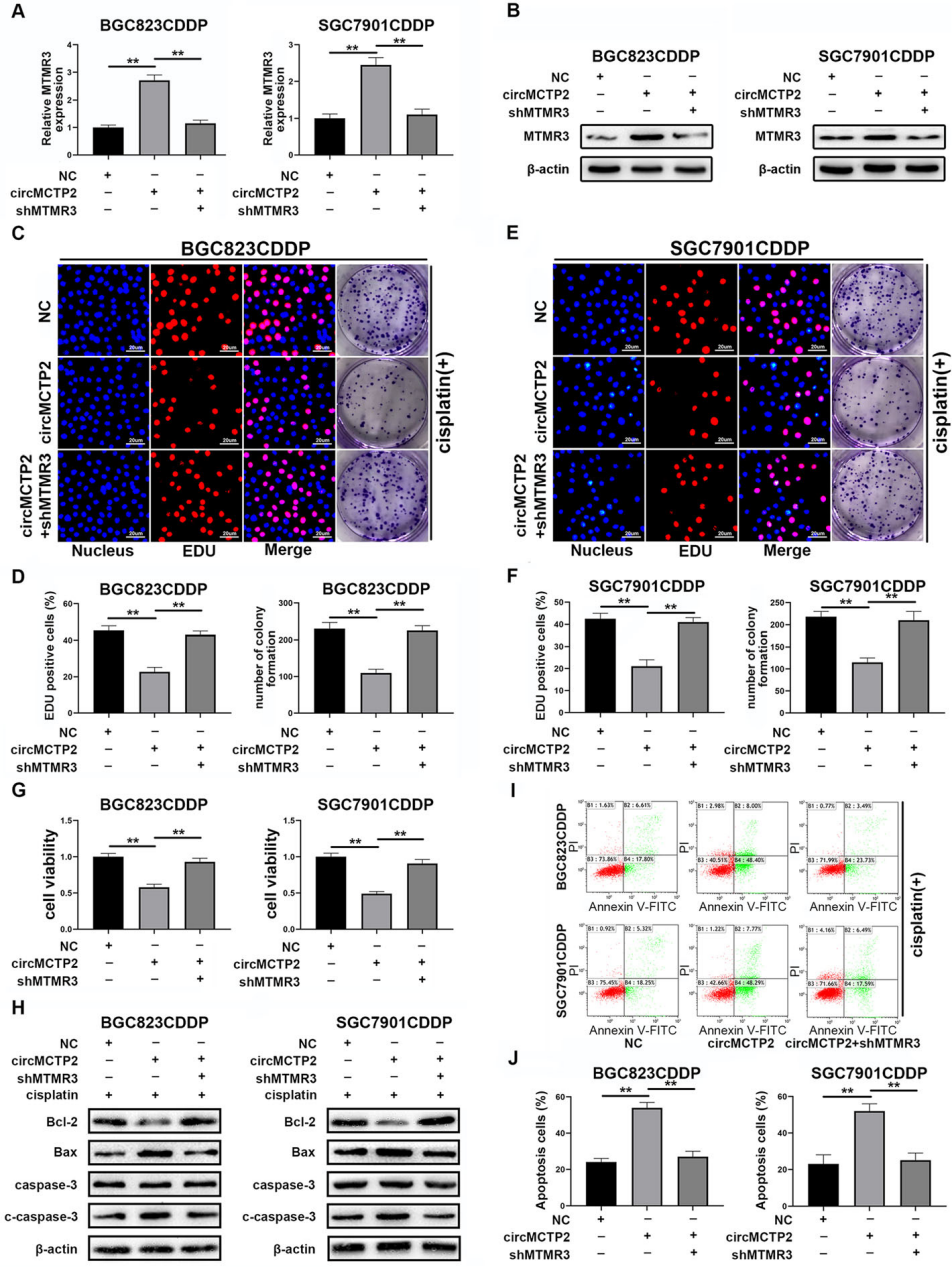
The effects of circMCTP2 can be reversed by the knockdown of MTMR3. **a** The expression levels of MTMR3 in BGC823CDDP and SGC7901CDDP cells after transfection with lentivirus-shMTMR3 were determined by qRT-PCR. **b** Protein expression levels of MTMR3 in BGC823CDDP and SGC7901CDDP cells were detected by western blotting. **c** and **d** Effects of circMCTP2 on cell proliferation and colony formation by BGC823CDDP cells were reversed by the knockdown of MTMR3. **e** and **f** Decreased cell proliferation and colony-forming ability of SGC7901CDDP cells caused by circMCTP2 were restored by the knockdown of MTMR3. **g** MTMR3 knockdown reversed the effects of circMCTP2 on cell viability. **h** Expression levels of apoptotic proteins were examined by western blotting. **i** and **j** Flow cytometric analysis was performed to determine whether shMTMR3 could reverse the effect of circMCTP2 on the apoptosis of BGC823CDDP and SGC7901CDDP cells. CDDP treatment: 12 μM for 48 h in BGC823CDDP cells and 6 μM for 48 h in SGC7901CDDP cells. (*p < 0.05, **p < 0.01. Data are expressed as the means ± SDs)

BGC823CDDP and SGC7901CDDP cells were transfected with lentivirus-shMTMR3 after circMCTP2 overexpression. MTMR3 expression levels were examined by qRT-PCR and western blotting (Fig. 6a and b). As shown in Fig. 6c and d, the decreased cell proliferation and colony forming ability of BGC823CDDP cells caused by circMCTP2 overexpression were restored by MTMR3 knockdown. Similar results were obtained for SGC7901CDDP cells (Fig. 6e and f). The influence of circMCTP2 on BGC823CDDP and SGC7901CDDP cell viability was rescued by the downregulation of MTMR3 (Fig. 6g). The results of flow cytometric analysis and western blotting indicated that the effect of circMCTP2 on BGC823CDDP and SGC7901CDDP cell apoptosis was rescued by MTMR3 knockdown (Fig. 6h-j). In summary, the downregulation of MTMR3 could counteract the effects of circMCTP2 on CDDP-resistant GC cells.

### The effect of circMCTP2 on autophagy is reversed by the downregulation of MTMR3

**Fig. 7:**
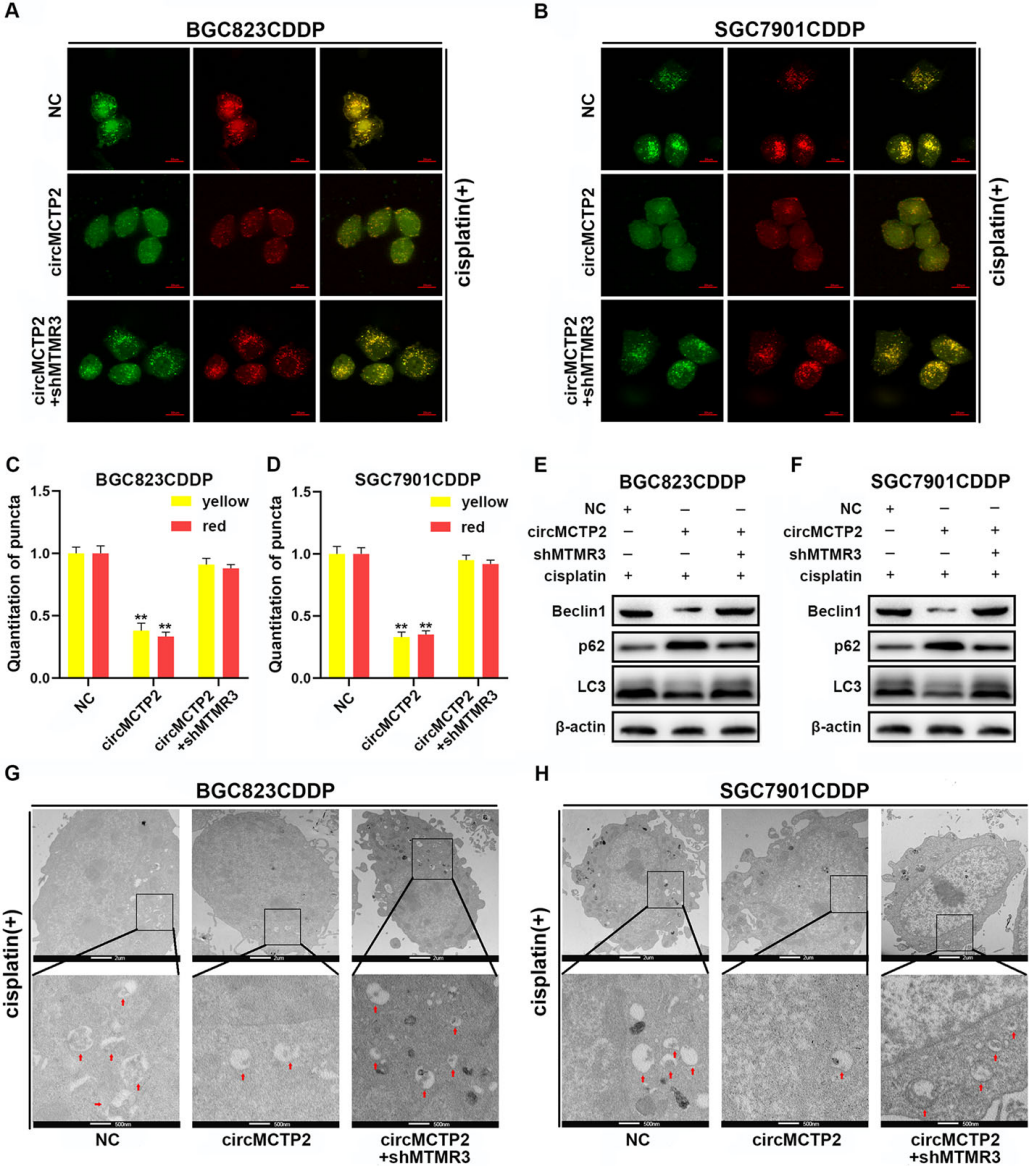
The effect of circMCTP2 on autophagy is reversed by the downregulation of MTMR3. **a-d** Confocal microscopy analysis showing the accumulation of GFP/mRFP-LC3 dots. **e** and **f** Western blotting showing the expression levels of Beclin1, p62, and LC3-II proteins. **g** and **h** TEM analysis showing the autophagic microstructure in CDDP-resistant GC cells. CDDP treatment: 12 μM for 48 h in BGC823CDDP cells and 6 μM for 48 h in SGC7901CDDP cells. (*p < 0.05, **p < 0.01. Data are expressed as the means ± SDs)

To examine whether the inhibitory effect of circMCTP2 on autophagy could be reversed by the knockdown of MTMR3, we performed confocal microscopy, TEM and western blotting. The reduced accumulation of GFP/mRFP-LC3 dots caused by circMCTP2 was reversed by the knockdown of MTMR3 in CDDP-resistant GC cells (Fig. 7a-d). We also observed that the decrease in protein expression of LC3-II caused by circMCTP2 overexpression was restored by knockdown of MTMR3 (Fig. 7e and f). Similar results were observed by TEM analysis (Fig. 7g and h). Taken together, downregulation of MTMR3 could counteract the effect of circMCTP2 on autophagy in CDDP-resistant GC cells.

### CircMCTP2 suppresses CDDP resistance in GC cells in vivo

**Fig. 8:**
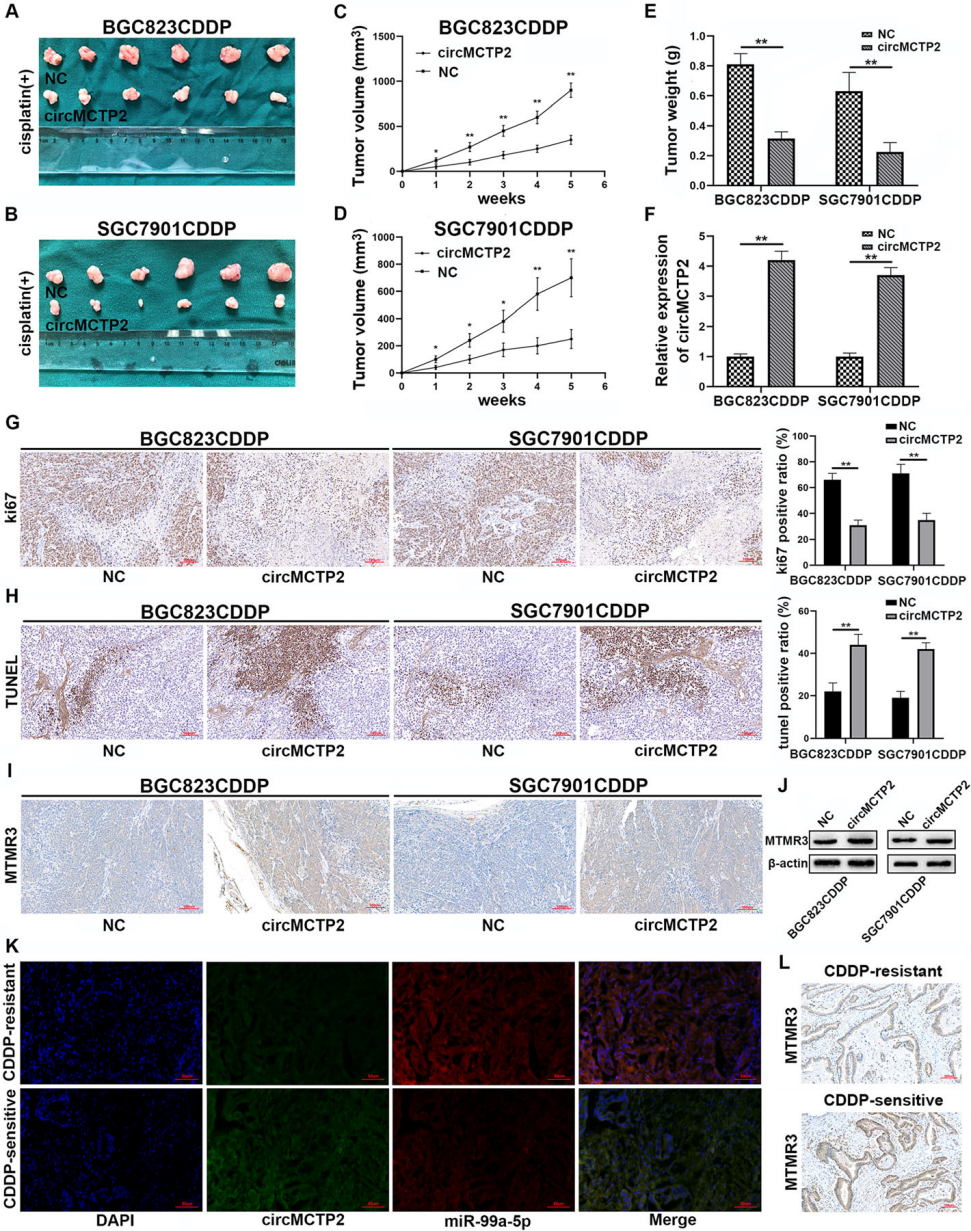
CircMCTP2 sensitizes GC cells to CDDP in vivo. **a-d** CircMCTP2 suppressed the growth of xenograft tumors with CDDP treatment. **e** Xenograft tumor weights were measured. **f** CircMCTP2 expression was detected by qRT-PCR in xenograft tumors. **g** The percentage of Ki67-positive cells in xenograft tumors was measured. **h** TUNEL assay was performed to determine the apoptosis of GC cells in nude mouse tumors. **i, j** MTMR3 protein expression in xenograft tumors was examined by IHC and western blotting. **k** The expression levels of circMCTP2 and miR-99a-5p were detected by FISH assay in CDDP-resistant and CDDP-sensitive GC tissues. **l** The results of IHC revealed that MTMR3 was downregulated in CDDP-resistant GC tissues. (*p < 0.05, **p < 0.01. Data are expressed as the means ± SDs)

To investigate the relationship between circMCTP2 and CDDP resistance in vivo, 1 × 10^6^ CDDP-resistant GC cells transfected with negative control (NC) or lentivirus-circMCTP2 were injected subcutaneously into each armpit of a nude mouse. CDDP (5 mg/kg) was injected intraperitoneally into nude mice three times per week. The proliferation rates of BGC823CDDP and SGC7901CDDP cells in vivo were reduced by the overexpression of circMCTP2 in response to CDDP treatment (Fig. 8a-d). It was also found that tumor weights were reduced by the overexpression of circMCTP2 (Fig. 8e). CircMCTP2 was overexpressed in the circMCTP2 groups compared to the NC groups (Fig. 8f). The IHC results suggested that the percentage of Ki67-positive cells was decreased by circMCTP2 (Fig. 8g). Additionally, the results of the TUNEL assay revealed that circMCTP2 promoted apoptosis of CDDP-resistant GC cells in vivo (Fig. 8h). MTMR3 expression was observed to be positively correlated with the expression of circMCTP2 in vivo by IHC and western blotting (Fig. 8i, j). FISH assays conducted on GC tissues further confirmed that circMCTP2 levels were lower in CDDP-resistant tissues than in CDDP-sensitive tissues, whereas miR-99a-5p expression showed the opposite trend (Fig. 8k). MTMR3 was confirmed to be downregulated in CDDP-resistant GC tissues by IHC (Fig. 8l).

## Discussion

Almost half of GC cases and GC-related deaths worldwide occur in China [32]. CDDP is one of the most widely used drugs for chemotherapy [33]. Chemoresistance is a major cause of chemotherapy failure in curing patients with cancer [34]. Understanding the mechanisms underlying resistance to CDDP in GC may contribute to a better treatment strategy for GC. Next-generation sequencing (NGS) was performed on CDDP-resistant and CDDP-sensitive GC cells to generate the circRNA profile. CircMCTP2 was selected because its expression levels were significantly different between the CDDP-resistant and CDDP-sensitive GC tissues. According to the follow-up data, patients with higher expression of circMCTP2 had a better prognosis. Analysis of the clinicopathological characteristics of the patients also revealed a correlation between circMCTP2 and CDDP resistance. We then carried out RNase R and actinomycin D assays to confirm the stable structure of circMCTP2. CircRNAs have rarely been reported to be associated with chemoresistance, and we are the first to determine the role of circMCTP2 in CDDP resistance in GC. In our study, we did not determine the cause of the downregulation of circMCTP2 in CDDP-resistant GC cells or tissues. It has been reported that RNA-binding proteins (RBPs), including DExH-Box Helicase 9 (DHX9), adenosine deaminase 1 acting on RNA (ADAR1) and quaking (QKI), are involved in the posttranscriptional formation of circRNAs [35–37]. Whether these three RBPs are involved in the downregulation of circMCTP2 remains uncertain. In our future research, we will try to explore the upstream regulatory mechanism of circMCTP2 in CDDP-resistant GC cells and tissues.

To explore the effect of circMCTP2 on CDDP resistance in GC, we first performed colony formation and EdU assays. The results suggested that the overexpression of circMCTP2 could inhibit the proliferation of CDDP-resistant GC cells in response to CDDP treatment. Flow cytometric analysis and western blotting were then performed, and the results indicated that upregulation of circMCTP2 could promote CDDP-resistant GC cell apoptosis. CircRNAs exert functions in many pathologies by RBPs [38], and some can even encode proteins [39]. However, miRNA sponging is the most reported mechanism by which circRNAs function in tumors [40, 41]. To predict the potential downstream miRNAs of circMCTP2, we combined the results of online databases and a microarray that was previously conducted to determine the miRNA profile of CDDP-resistant GC cells. By RNA pull-down assay, miR-99a-5p was then selected as the possible miRNA that could be sponged by circMCTP2. Finally, we performed a luciferase reporter assay to confirm the binding between miR-99a-5p and circMCTP2. The FISH assay results revealed that both circMCTP2 and miR-99a-5p localized to the cytoplasm of CDDP-resistant GC cells. MiR-99a-5p was validated to contribute to CDDP resistance by colony formation assay, EdU assay, flow cytometric analysis, and western blotting, which was consistent with a previously published study [16]. MTMR3 was determined by online databases to be downstream of miR-99a-5p, which was supported by the results of luciferase reporter, pull-down, and Ago2 RIP assays. CircMCTP2 was shown to upregulate MTMR3 by sponging miR-99a-5p in qRT-PCR and western blotting assays. To further explore whether knockdown of MTMR3 could reverse the effect of circMCTP2, rescue experiments were carried out. The reduced proliferation and increased apoptosis of CDDP-resistant GC cells caused by circMCTP2 were counteracted by the knockdown of MTMR3.

Autophagy has been reported to act as both a tumor promoter and tumor suppressor in carcinogenesis [42, 43]. Autophagy functions as a barrier to tumor initiation, while it promotes cancer progression and maintenance after neoplastic lesions have been established [44]. Autophagy can provide substrates to help tumor cells overcome nutrient limitations and hypoxia [45]. Autophagy has also been elucidated to promote resistance to chemotherapy in tumors [46]. MTMR3 was confirmed to reduce autophagic activity by acting as a PI3P phosphatase [25]. By IHC and western blotting, we found that P62, which is negatively related to autophagy, was downregulated in CDDP-resistant GC tissues. We further detected autophagy in CDDP-resistant GC cells by confocal microscopy, TEM, and western blotting. The results of these experiments showed that autophagy was inhibited by the overexpression of circMCTP2. The inhibitory effect of circMCTP2 on autophagy was also found to be reversed by the knockdown of MTMR3. CDDP has direct cytotoxicity to tumor cells by binding to nuclear DNA [47, 48]. Eukaryotic and prokaryotic cells are hypersensitive to CDDP due to deficiencies in DNA repair [49]. An enhanced DNA repair ability in tumor cells results in resistance to CDDP and subsequent tumor recurrence [50]. Inhibition of autophagy might be one of the mechanisms by which circMCTP2 suppresses CDDP resistance in GC cells. More research is needed in the future to investigate whether DNA repair is strengthened in CDDP-resistant GC cells and how circMCTP2 affects DNA repair.

To examine the influence of circMCTP2 on CDDP resistance in vivo, a xenograft tumor model was established with nude mice. CDDP was injected intraperitoneally three times per week according to the weight of the nude mouse. The volumes of the tumors with higher expression of circMCTP2 were found to be smaller than those of the control group. Based on the results of IHC and TUNEL assays, circMCTP2 could also suppress CDDP resistance in vivo.

There are limitations to this study. The expression of circMCTP2 was evaluated only in human GC tissues. Circulating circRNAs are reportedly better biomarkers for certain pathologies [51, 52]. In this study, plasma samples of patients with GC were not collected, and thus, we failed to determine whether plasma circMCTP2 could be a suitable biomarker to distinguish patients with CDDP-resistant GC from those with CDDP-sensitive GC. CircMCTP2 was demonstrated only to function as a miRNA sponge, and whether circMCTP2 could regulate chemoresistance by binding to RBPs was not explored. MTMR3 was one of the targets of miR-99a-5p, and we could not rule out the possibility that there might be other target genes. *Helicobacter pylori* has been shown to be one of the most important pathogenic factors for GC [53]. It has also been reported that *H. pylori* can regulate CDDP resistance [54, 55]. Nevertheless, many of the patients were not examined for *H. pylori*, so we did not analyze the relationship between *H. pylori* and circMCTP2.

## Conclusion

CircMCTP2 has been demonstrated to be aberrantly downregulated in CDDP-resistant GC cells and tissues. Overexpression of circMCTP2 can sensitize GC cells to CDDP by sponging miR-99a-5p to restore the expression of MTMR3. Our findings may provide novel insights to counteract CDDP resistance during chemotherapy.

## Supplementary Information

**Additional file 1:** **Table S1.** Primers for qRT-PCR in this study.

**Additional file 2: Fig. S1.** CircMCTP2 is a predictive biomarker for CDDP resistance in GC and a favorable factor for prognosis of GC patients. (a) Expression of circMCTP2 in 75 CDDP-sensitive and 25 CDDP-resistant GC tissues. **(b)** ROC curve of circMCTP2 with the area under the curve being 0.9450. **(c, d)** Kaplan-Meier survival curves of DFS and OS for patients with high (*n* = 50) or low (*n* = 50) expression of circMCTP2. The median circMCTP2 expression value was used as the cutoff. (*p < 0.05, **p < 0.01. Data are expressed as the means ± SDs).

**Additional file 3: Fig. S2.** Lentivirus transfection efficiency was detected by qRT-PCR in CDDP-resistant GC cells. (a, b) The expression of circMCTP2 and MCTP2 mRNA after lentivirus-circMCTP2 transfection by qRT-PCR is shown. **(c, d)** The transfection efficiency of lentivirus-miR-99a-5p inhibitor was determined by qRT-PCR in BGC823CDDP and SGC7901CDDP cells. (*p < 0.05, **p < 0.01. Data are expressed as the means ± SDs).

**Additional file 4: Fig. S3.** MiR-99a-5p is not digested by cirMCTP2. (a) Overexpression of circMCTP2 had no effect on the expression level of miR-99a-5p in CDDP-resistant GC cells. **(b)** There was no linear correlation between the expression levels of circMCTP2 and miR-99a-5p in CDDP-resistant GC tissues. (*p < 0.05, **p < 0.01. Data are expressed as the means ± SDs).

**Additional file 5: Fig. S4.** Knockdown of miR-99a-5p sensitizes GC cells to CDDP. (a) The number of colonies formed by BGC823CDDP and SGC7901CDDP cells was reduced after miR-99a-5p knockdown. **(b)** DNA synthesis in BGC823CDDP and SGC7901CDDP cells was repressed by miR-99a-5p inhibition. **(c)** Apoptosis of CDDP-resistant GC cells transfected with miR-NC or miR-99a-5p inhibitor was detected by flow cytometric analysis. **(d)** The effect of miR-99a-5p on the apoptosis of BGC823CDDP and SGC7901CDDP cells was examined by western blotting. CDDP treatment: 12 μM for 48 h in BGC823CDDP cells and 6 μM for 48 h in SGC7901CDDP cells. (*p < 0.05, **p < 0.01. Data are expressed as the means ± SDs).

## Data Availability

All data in our study will be available upon reasonable request.

## Abbreviations

qRT-PCR: Quantitative real-time polymerase chain reaction
CDDP: Cisplatin
circRNA: Circular RNA
miRNA: MicroRNA
GC: Gastric cancer
3′-UTR: 3′-Untranslated region
MTMR3: Myotubularin-related protein 3
PI3P: PtdIns3P
FISH: Fluorescence in situ hybridization
TEM: Transmission electron microscopy
DFS: Disease-free survival
RIP: RNA Immunoprecipitation
Bio-miR-99a-5p: Biotinylated-miR-99a-5p
Bio-NC: Biotinylated-miR-NC
OS: Overall survival
gDNA: Genomic DNA
IHC: Immunohistochemistry
GEO: Gene expression omnibus
NGS: Next-generation sequencing
RBP: RNA-binding protein
SD: Standard deviation
DHX9: DExH-box helicase 9
ADAR1: Adenosine deaminase 1 acting on RNA
QKI: Quaking
