# Supplementary material for: Circular RNA MCTP2 inhibits cisplatin resistance in gastric cancer by miR-99a-5p-mediated induction of MTMR3 expression
Source: J Exp Clin Cancer Res. 2020 Nov 17;39:246. doi: 10.1186/s13046-020-01758-w (PMC7670601; doi:10.1186/s13046-020-01758-w)
Supplement: Supplementary file 1 — Additional file 1: Table S1. Primers for qRT-PCR in this study. [file 13046_2020_1758_MOESM1_ESM.pdf]

Additional file 1: Table S1

hsa_circ_0000657 (circMCTP2)-Forward	ACCAGAAGAGCCAGAGGAGTC
hsa_circ_0000657 (circMCTP2)-Reverse	TGGCCTGGTCCGCTGTTTTAA
hsa_circ_0000659-Forward	GCGGCAGATTTCTCAGTCCTC
hsa_circ_0000659-Reverse	CCCGTTCCAGAGTTGGTTCTT
hsa_circ_0037012-Forward	CCGTCTGATGTTCAAAAGGAATC
hsa_circ_0037012-Reverse	GCTATTTCCTCCAAGACGTTTTG
hsa_circ_0008591-Forward	CATCTGCTACATCCTAGCCGAA
hsa_circ_0008591-Reverse	GTCACTGTCCGGTAATCAACCTC
hsa_circ_0082374-Forward	ATTTGTACAGCATCTGGTGTGTGC
hsa_circ_0082374-Reverse	GTCTTCCTCCATAATCGGTTTGAA
hsa_circ_0067047-Forward	TCGTCATGGTCCTGGAGATAGC
hsa_circ_0067047-Reverse	TAATCAACCTCGGGCTCCTTCT
miR-99a-5p-Forward	AACCCGTAGATCCGATCTTGTG
miR-324-5p-Forward	CGCATCCCCTAGGGCATTGGTG
miR-485-5p-Forward	AGAGGCTGGCCGTGATGAATTC
miR-149-5p-Forward	TCTGGCTCCGTGTCTTCACTCCC
miR-708-5p-Forward	AAGGAGCTTACAATCTAGCTGGG
miR-452-5p-Forward	AACTGTTTGCAGAGGAAACTGA
miR-188-5p-Forward	CATCCCTTGCATGGTGGAGGG
miR-1285-3p-Forward	TCTGGGCAACAAAGTGAGACCT
Universal 5' primer	GCGAGCACAGAATTAATACGAC
MCTP2-Forward	CAGATCTACGGGCAAGGCAT
MCTP2-Reverse	GCACCGAGGTGTAGGAAGAC
MTMR3-Forward	AGCAGAGTGGGCTCAGTGTT
MTMR3-Reverse	ACTGTCCACGTTTGGTCCTC
β-actin-Forward	CTCCATCCTGGCCTCGCTGT
β-actin-Reverse	GCTGTCACCTTCACCGTTCC
U6-Forward	CTCGCTTCGGCAGCACA
U6-Rerverse	AACGCTTCACGAATTTGCGT
