# Supplementary figures and images for: Circular RNA MCTP2 inhibits cisplatin resistance in gastric cancer by miR-99a-5p-mediated induction of MTMR3 expression
Source: J Exp Clin Cancer Res. 2020 Nov 17;39:246. doi: 10.1186/s13046-020-01758-w (PMC7670601; doi:10.1186/s13046-020-01758-w)

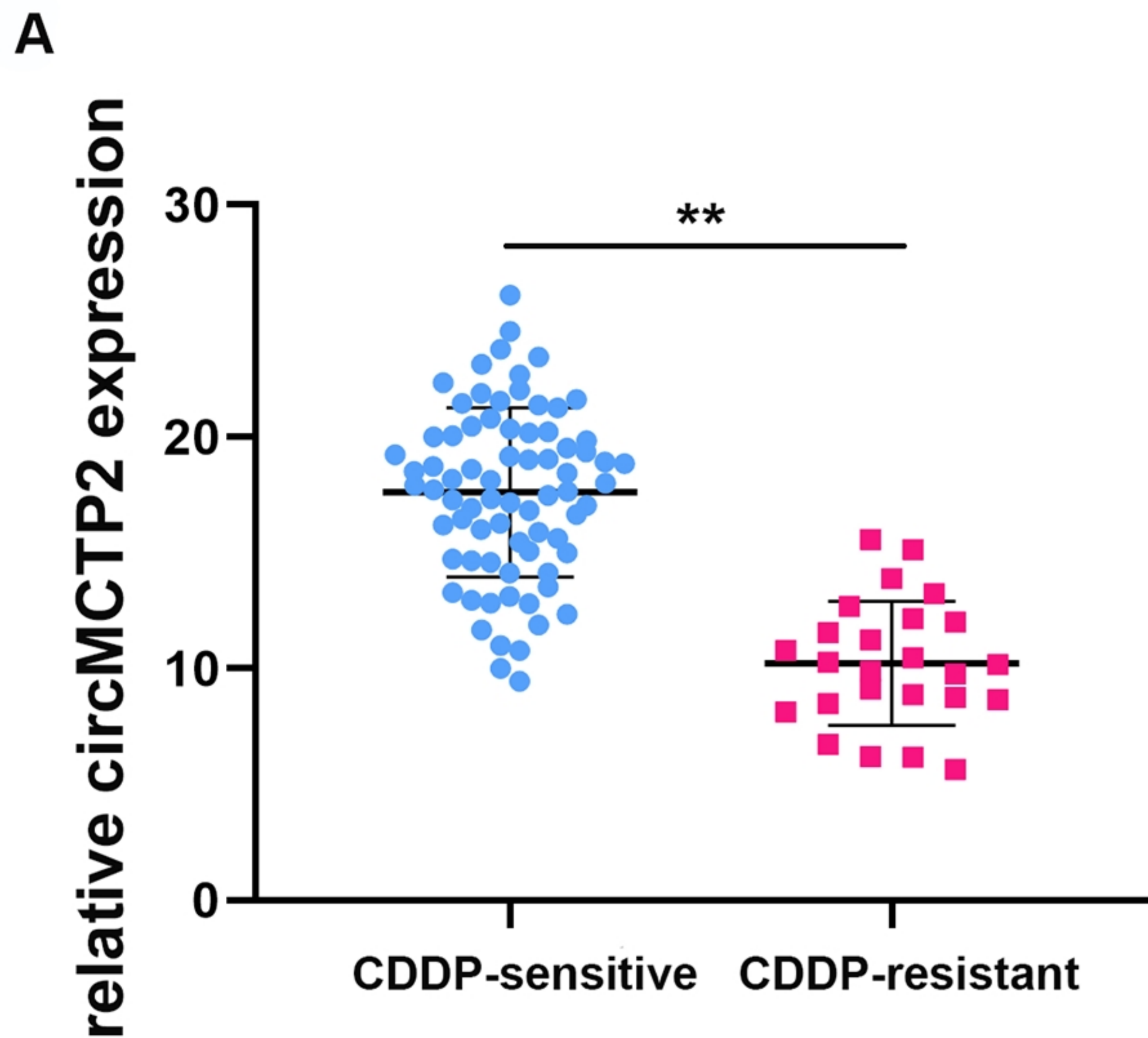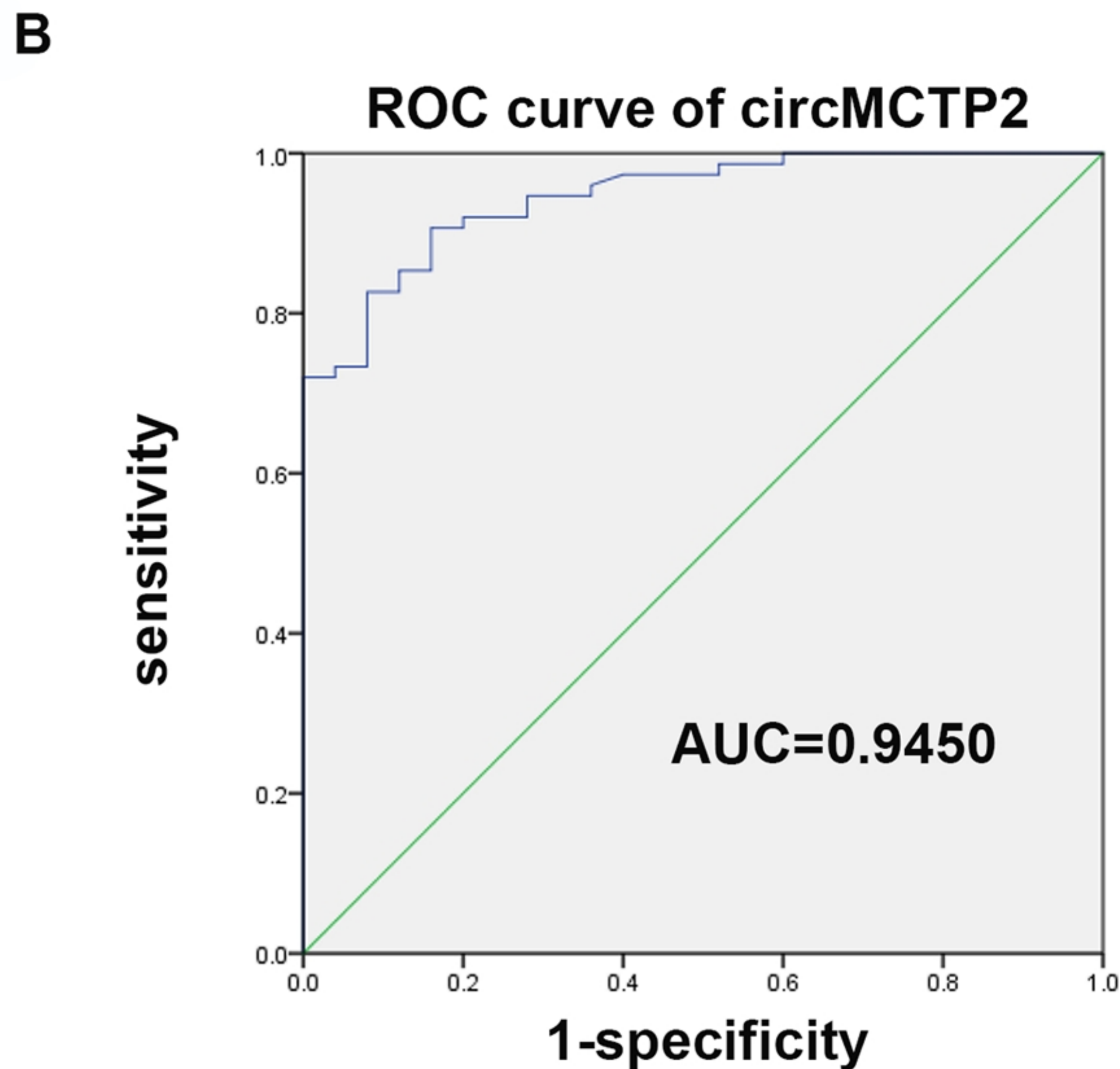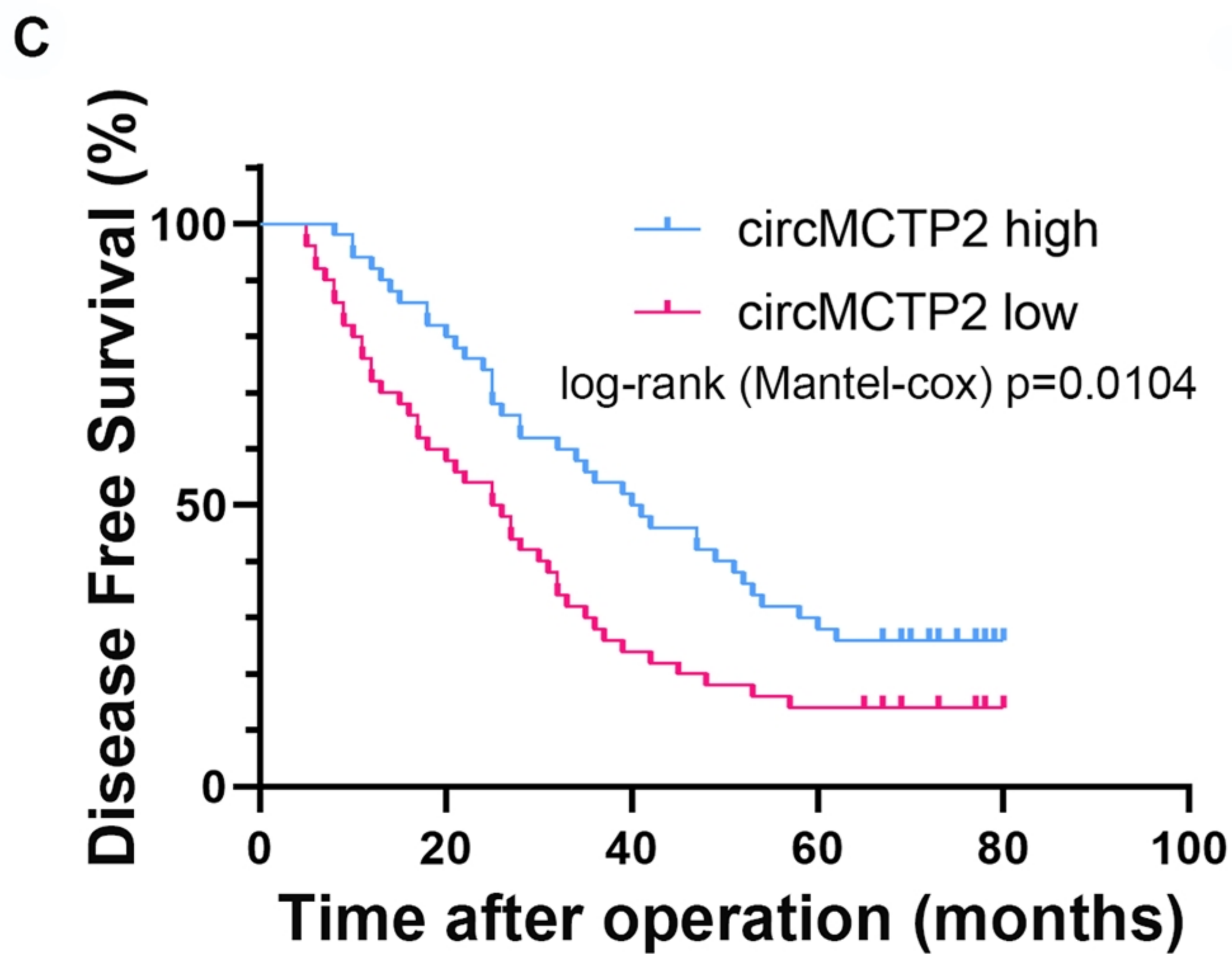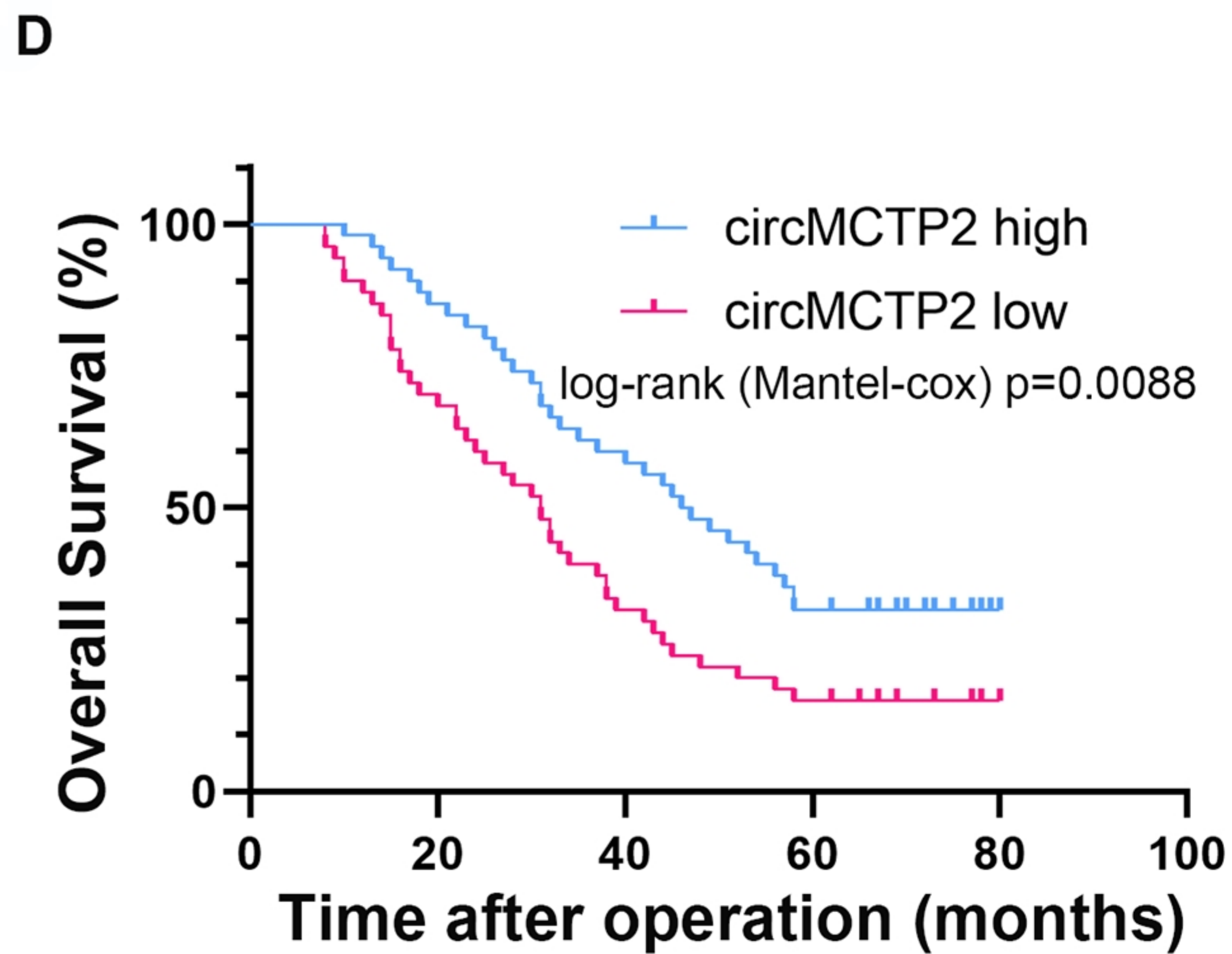

Supplement: Supplementary file 2 — Additional file 2: Fig. S1. CircMCTP2 is a predictive biomarker for CDDP resistance in GC and a favorable factor for prognosis of GC patients. (a) Expression of circMCTP2 in 75 CDDP-sensitive and 25 CDDP-resistant GC tissues. (b) ROC curve of circMCTP2 with the area under the curve being 0.9450. (c, d) Kaplan-Meier survival curves of DFS and OS for patients with high (n = 50) or low (n = 50) expression of circMCTP2. The median circMCTP2 expression value was used as the cutoff. (*p < 0.05, **p < 0.01. Data are expressed as the means ± SDs). [file 13046_2020_1758_MOESM2_ESM.pdf]

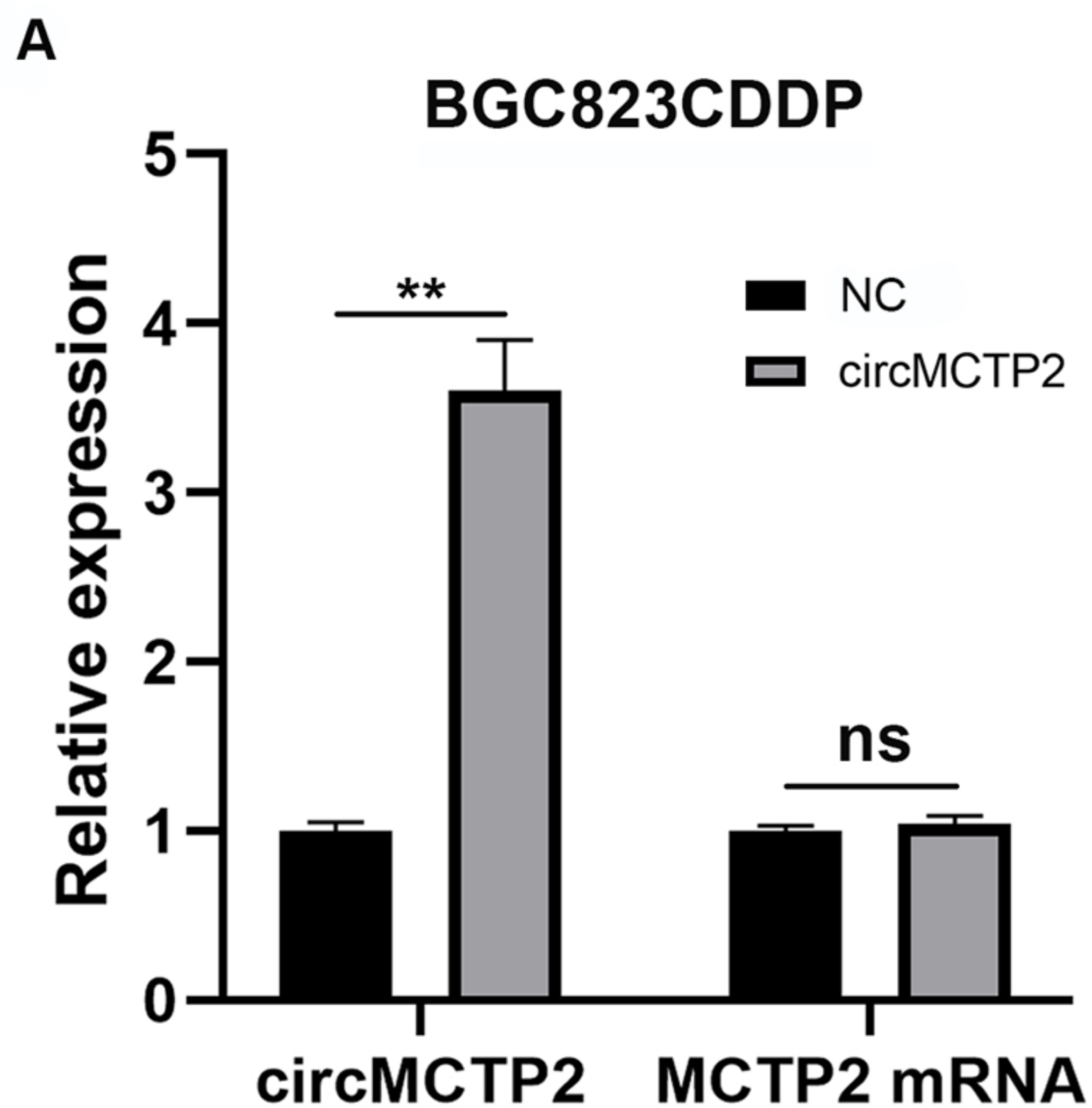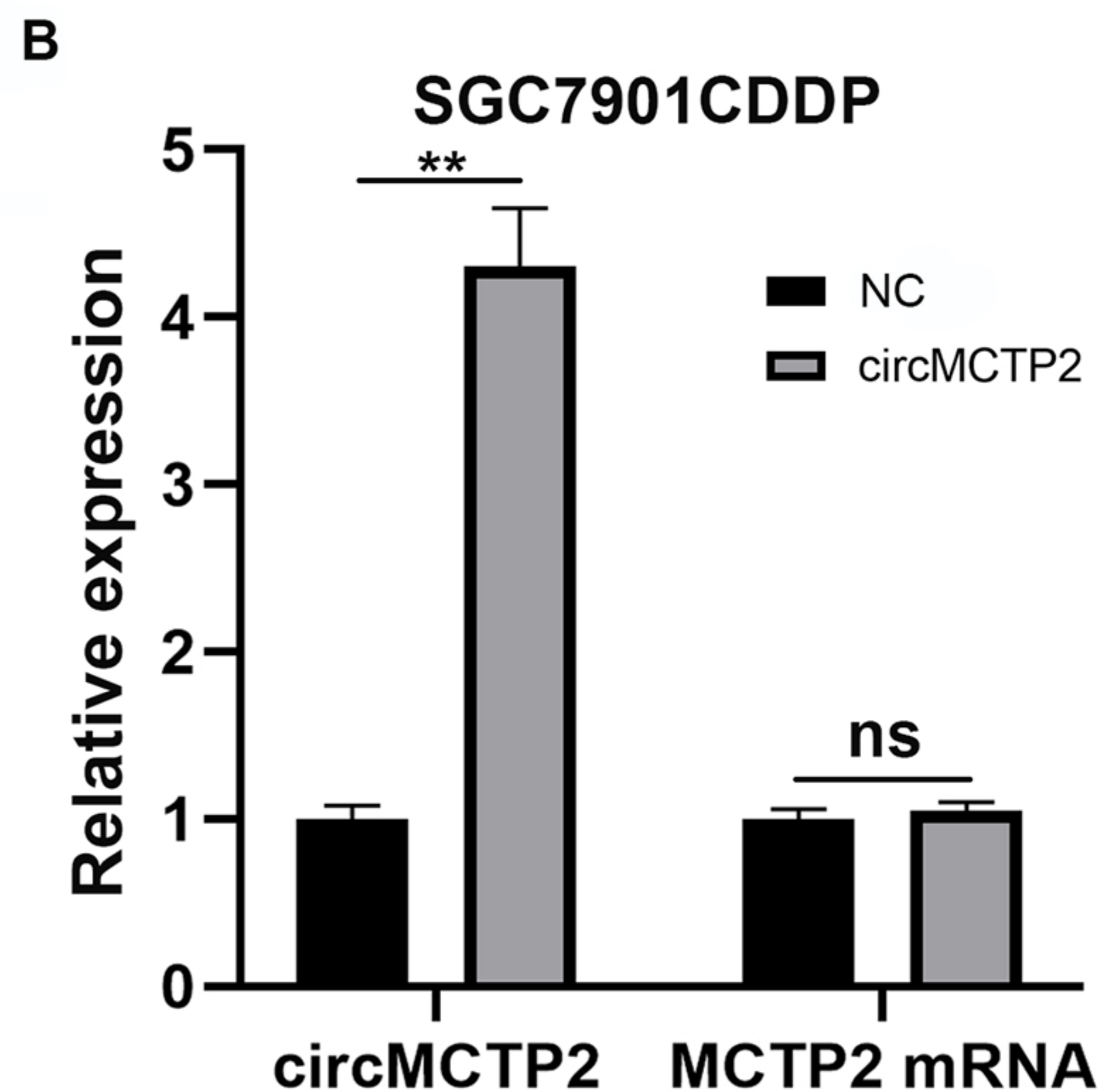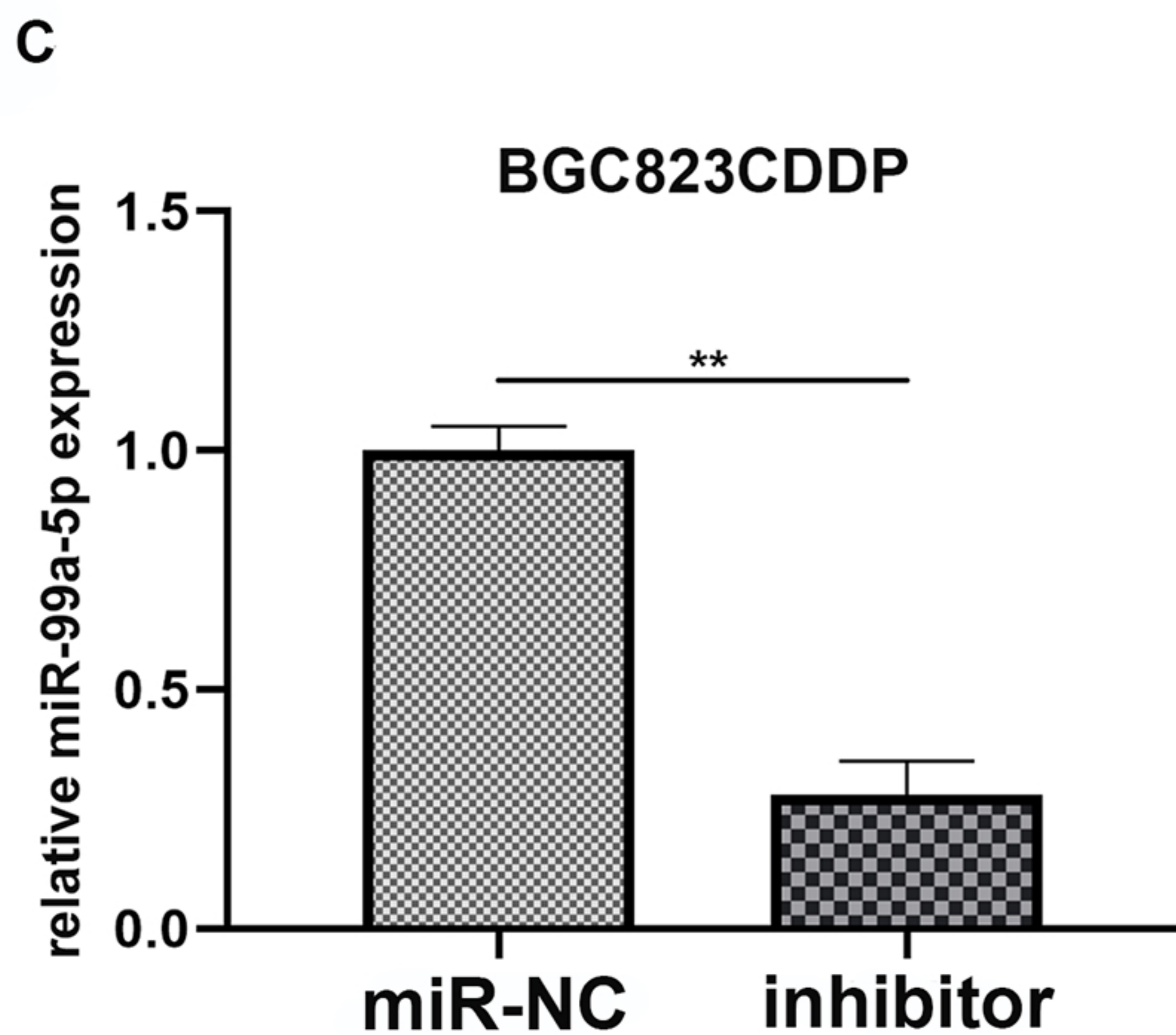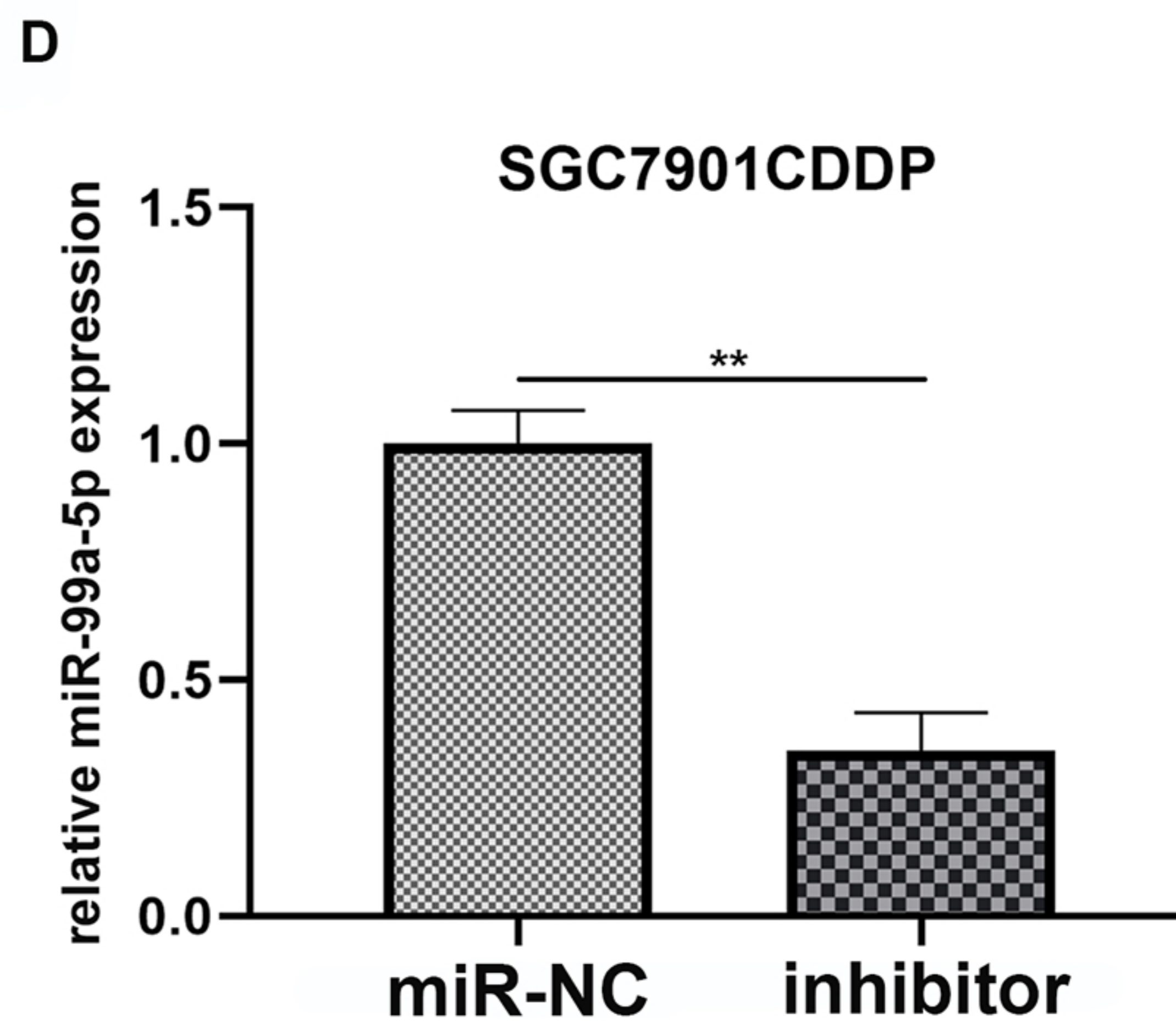

Supplement: Supplementary file 3 — Additional file 3: Fig. S2. Lentivirus transfection efficiency was detected by qRT-PCR in CDDP-resistant GC cells. (a, b) The expression of circMCTP2 and MCTP2 mRNA after lentivirus-circMCTP2 transfection by qRT-PCR is shown. (c, d) The transfection efficiency of lentivirus-miR-99a-5p inhibitor was determined by qRT-PCR in BGC823CDDP and SGC7901CDDP cells. (*p < 0.05, **p < 0.01. Data are expressed as the means ± SDs). [file 13046_2020_1758_MOESM3_ESM.pdf]

**A**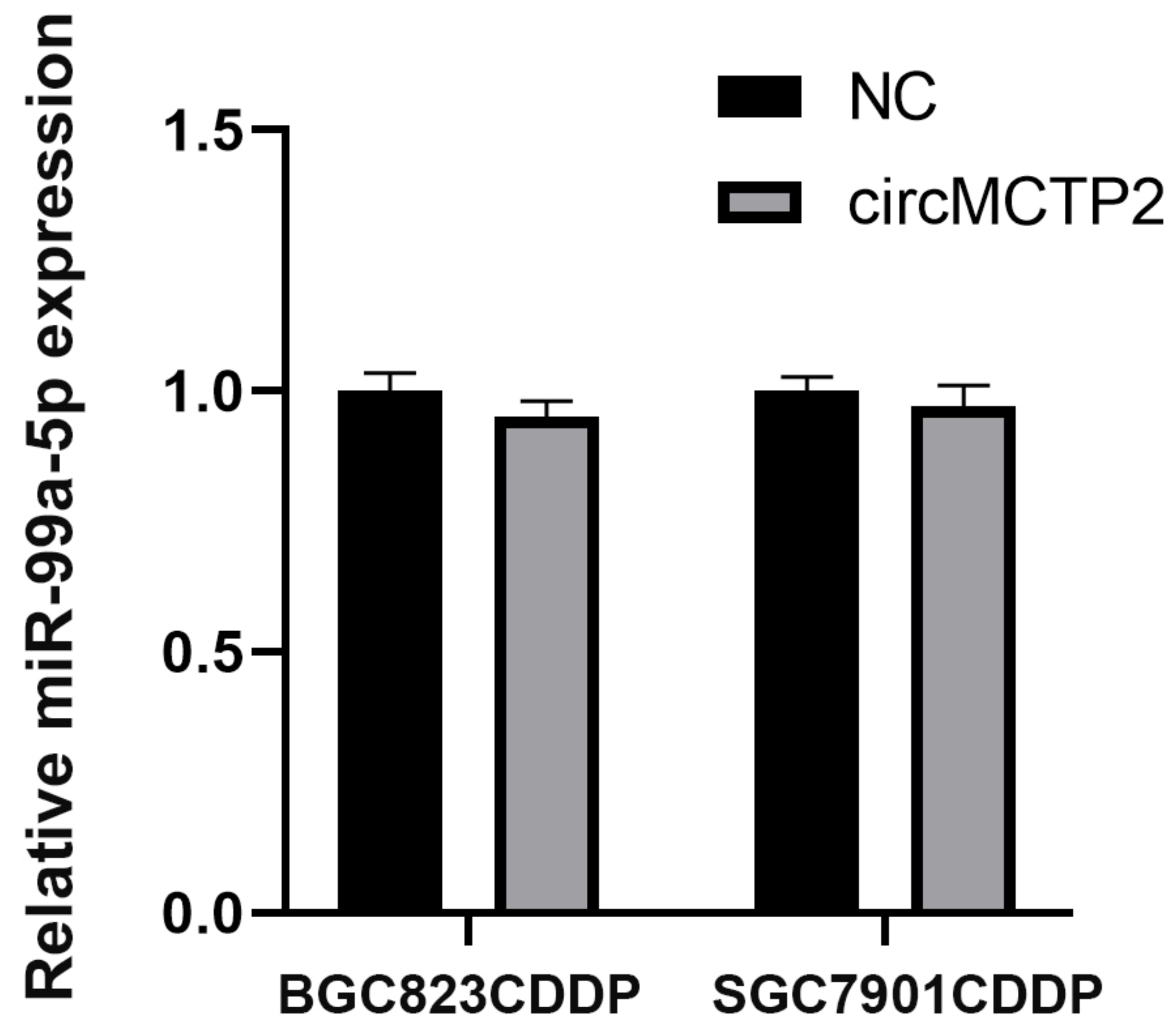**B**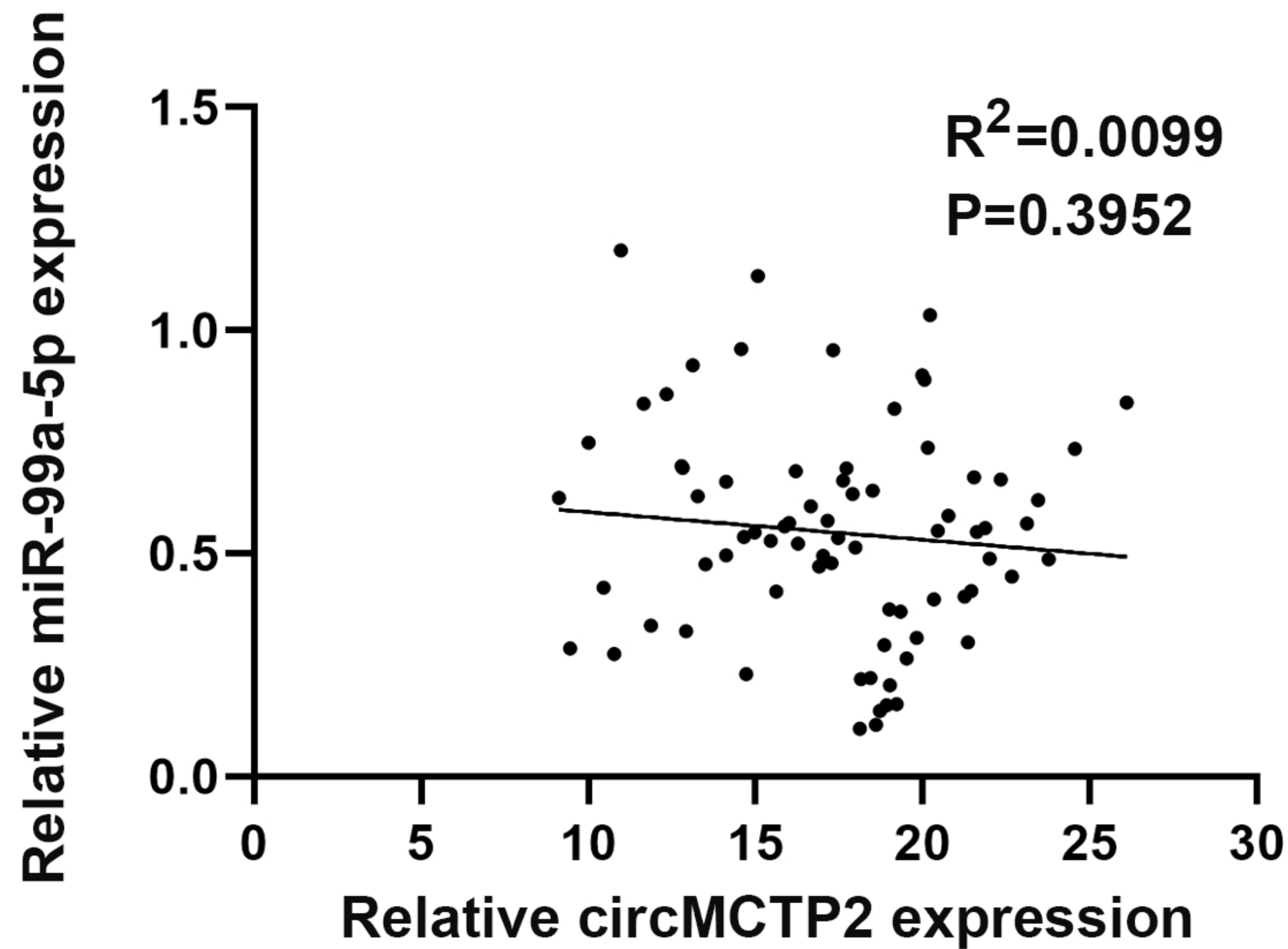

Supplement: Supplementary file 4 — Additional file 4: Fig. S3. MiR-99a-5p is not digested by cirMCTP2. (a) Overexpression of circMCTP2 had no effect on the expression level of miR-99a-5p in CDDP-resistant GC cells. (b) There was no linear correlation between the expression levels of circMCTP2 and miR-99a-5p in CDDP-resistant GC tissues. (*p < 0.05, **p < 0.01. Data are expressed as the means ± SDs). [file 13046_2020_1758_MOESM4_ESM.pdf]

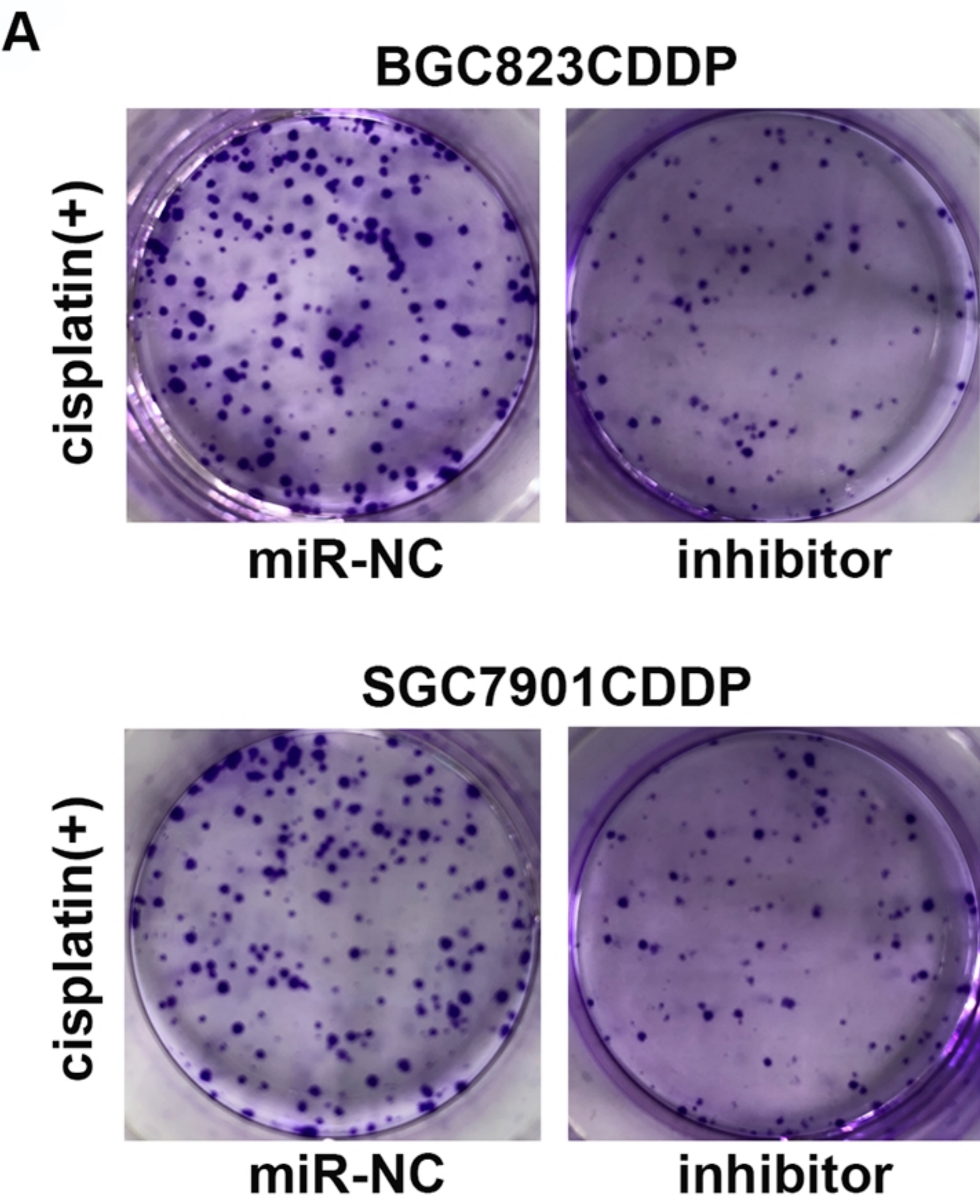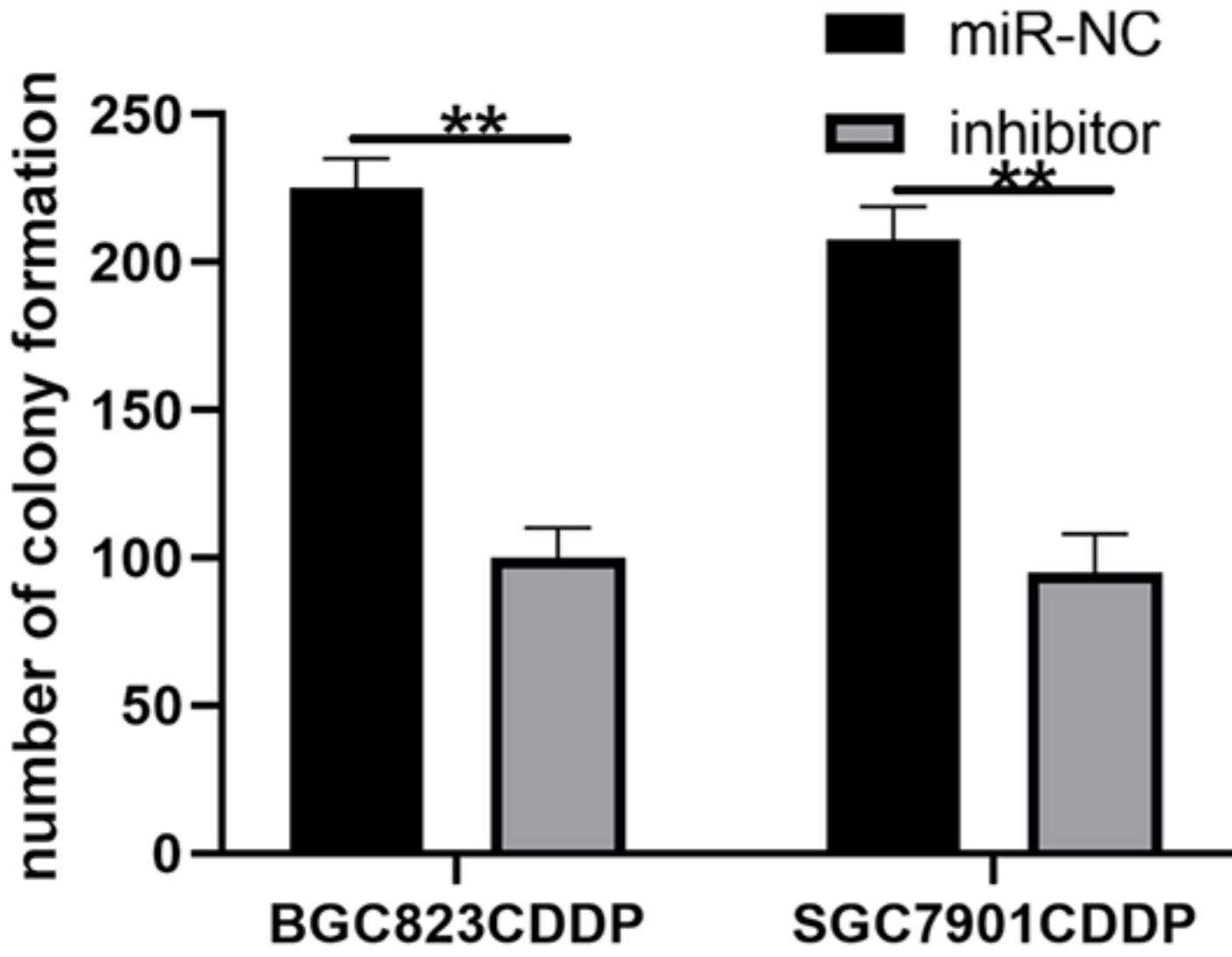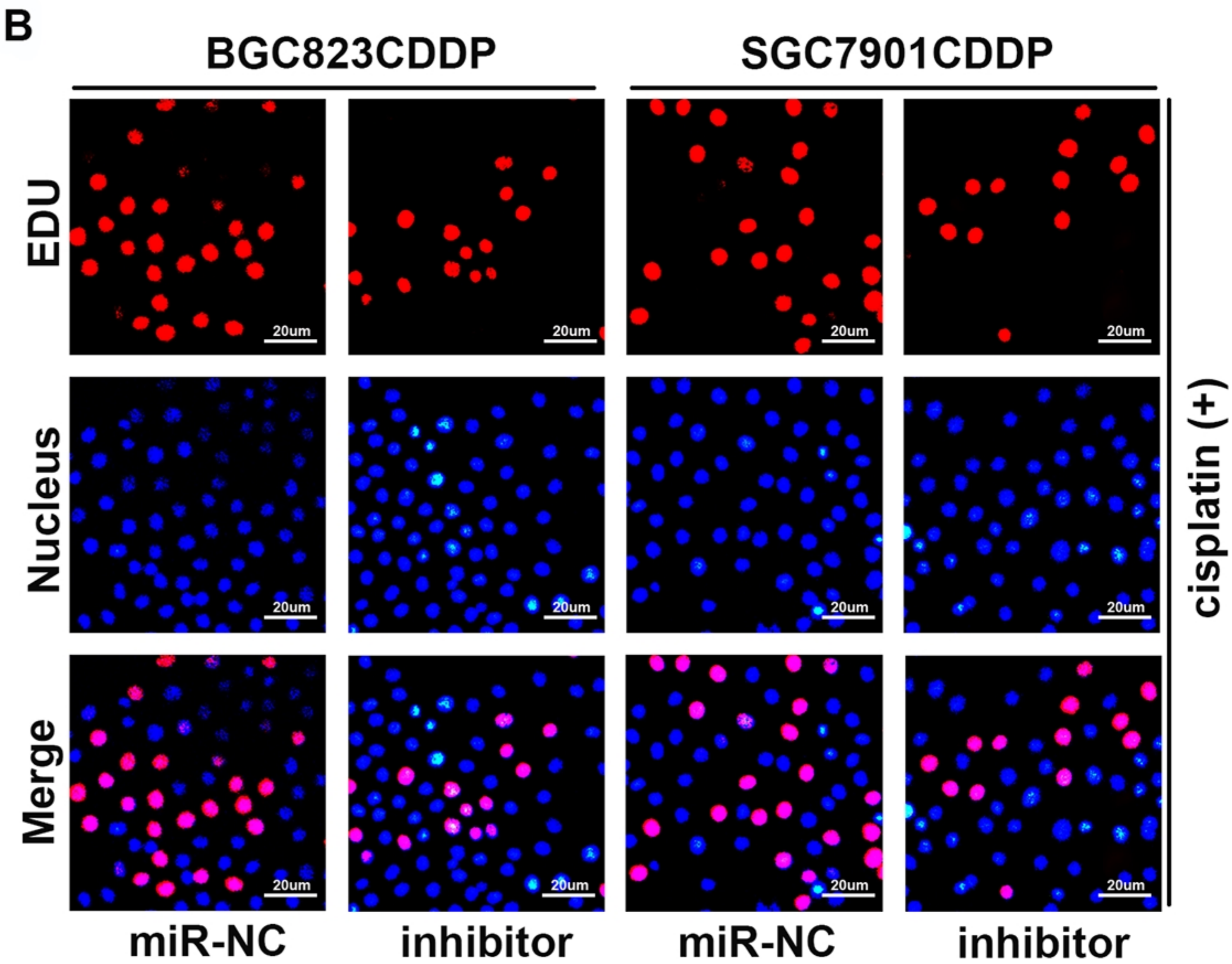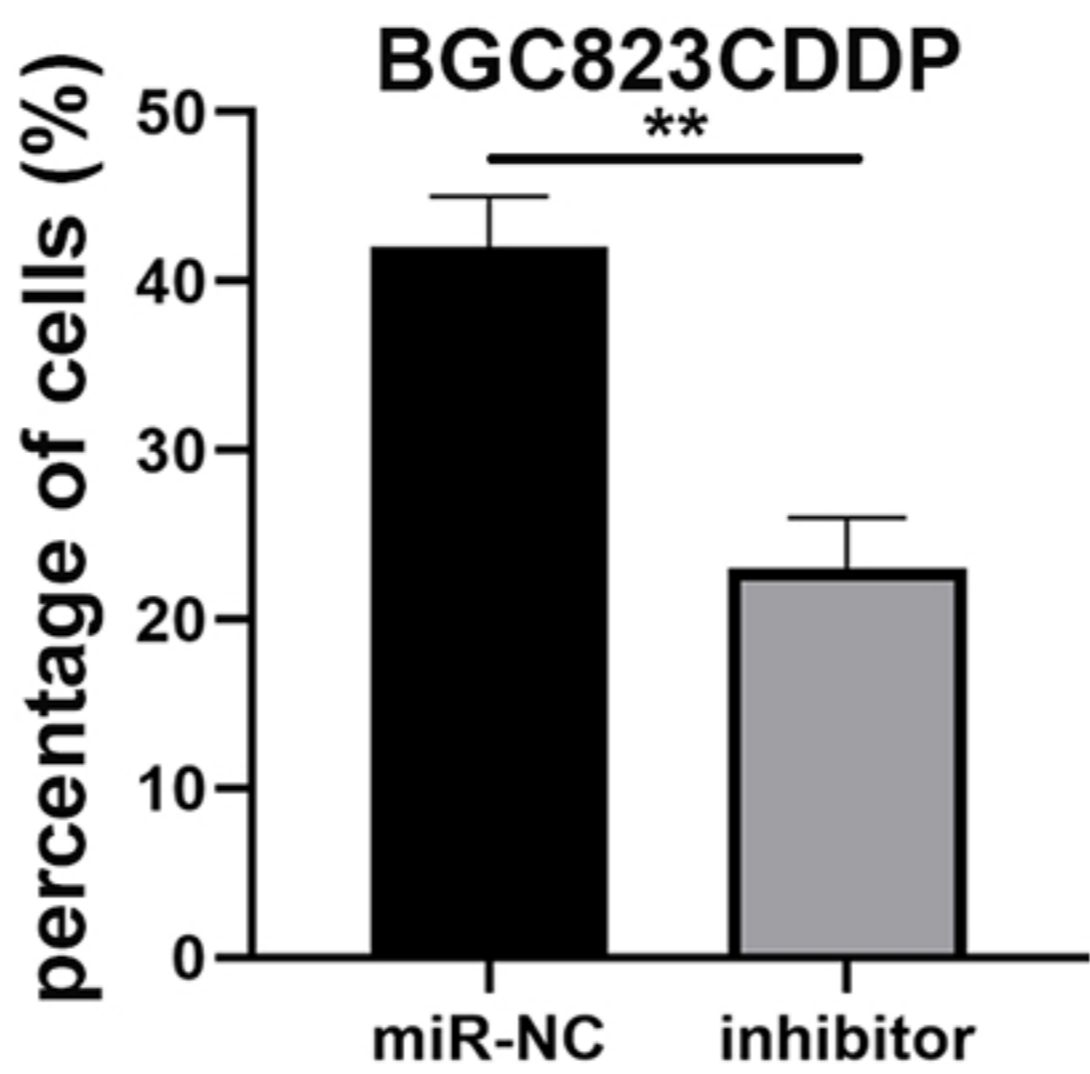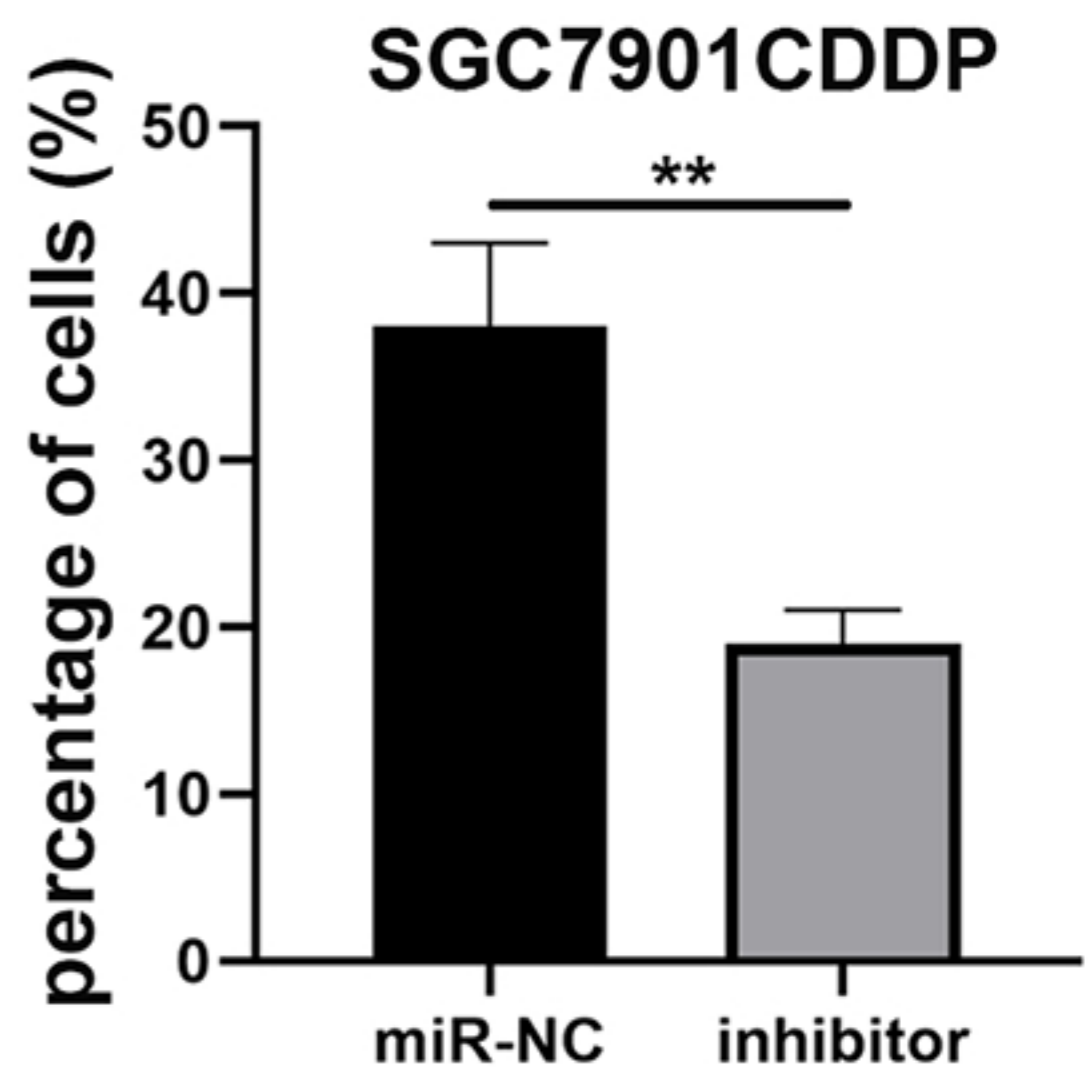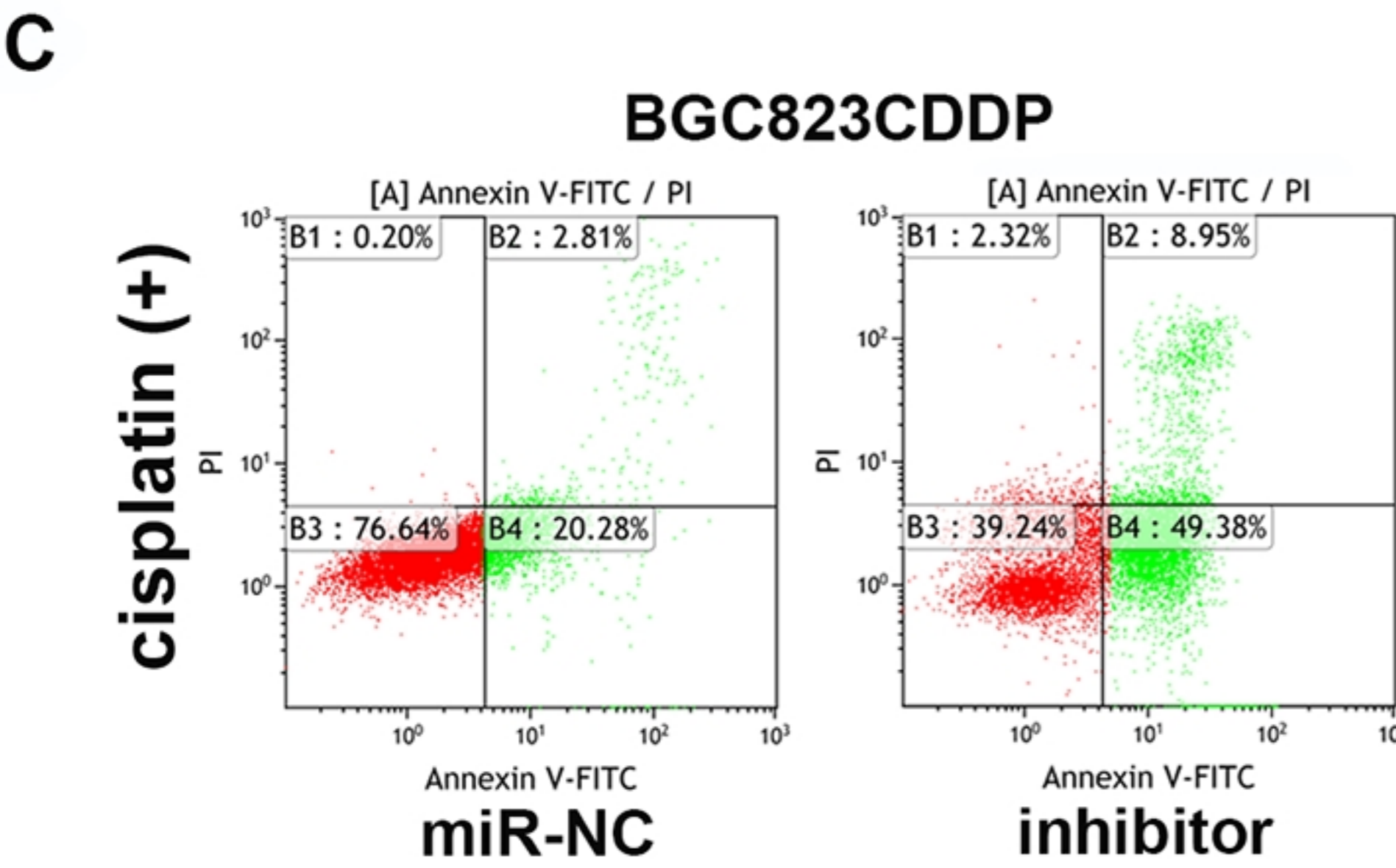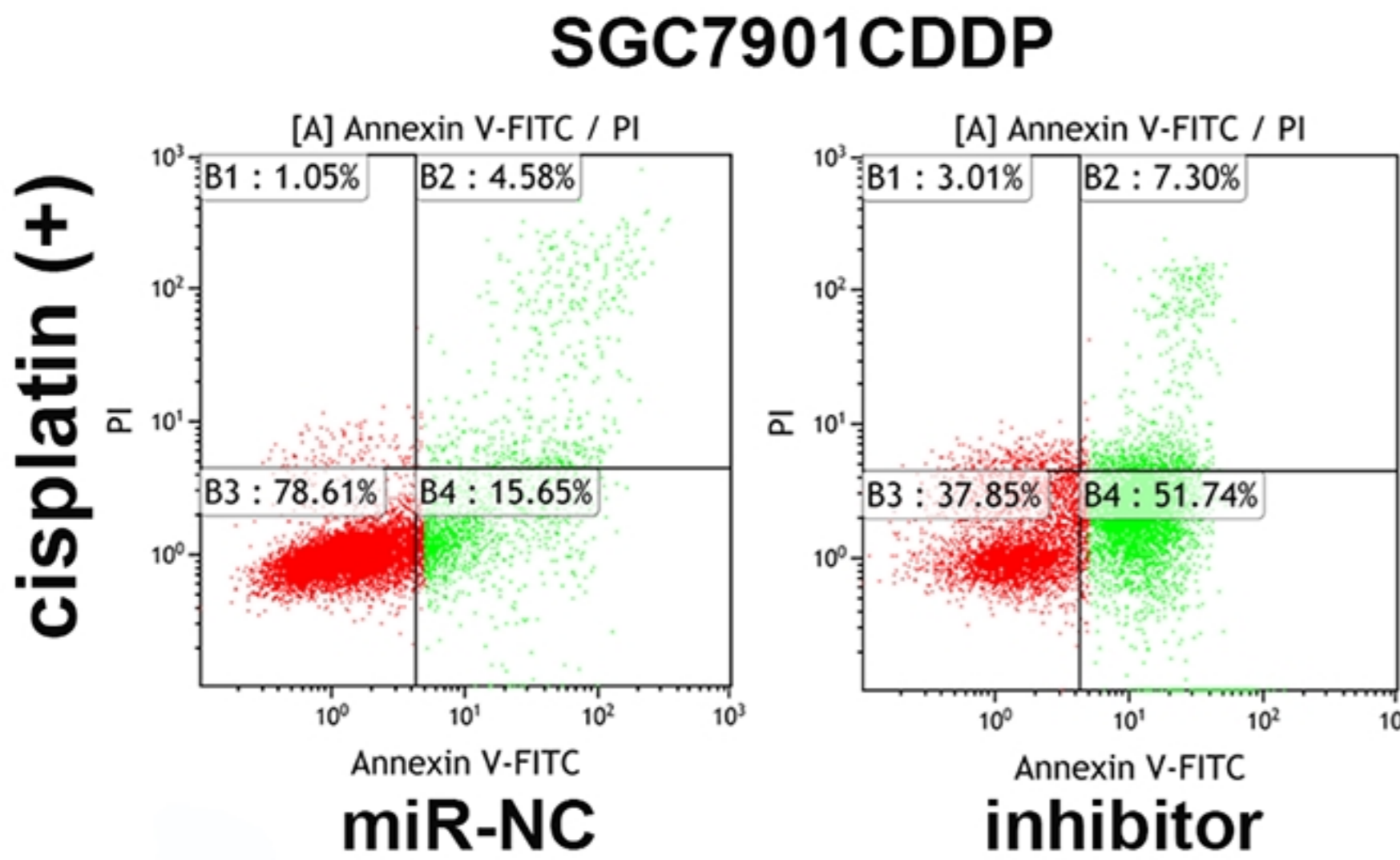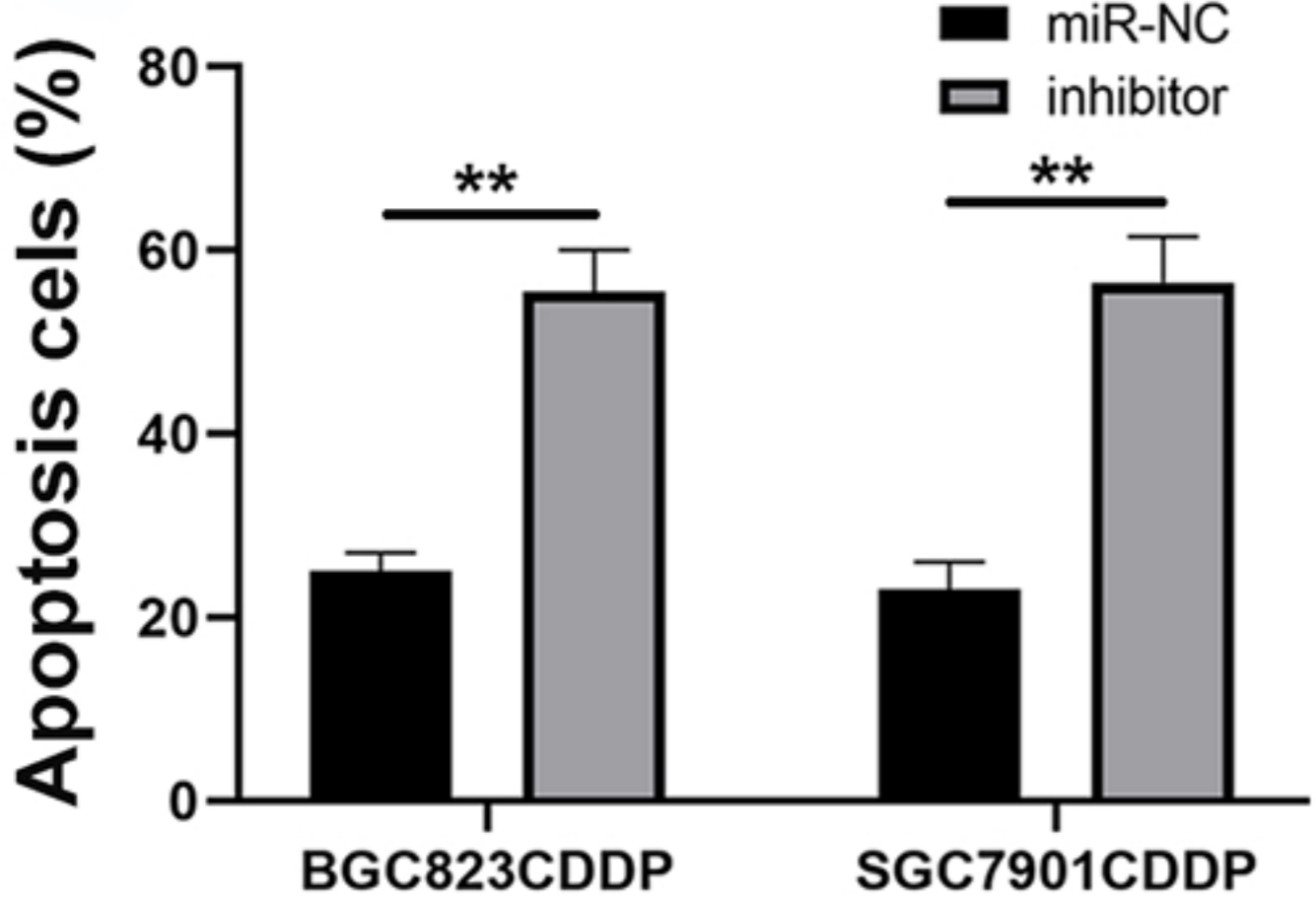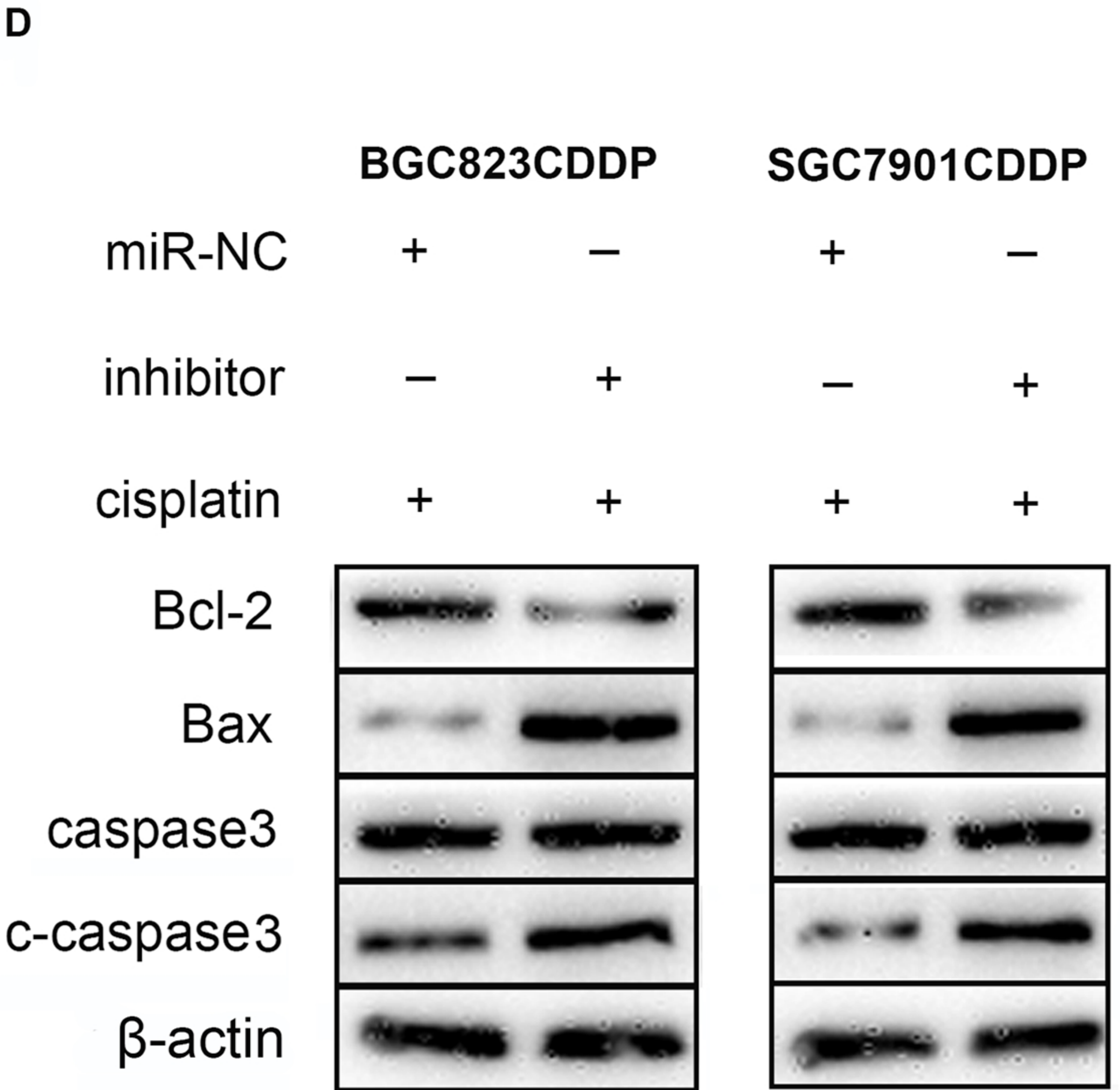

Supplement: Supplementary file 5 — Additional file 5: Fig. S4. Knockdown of miR-99a-5p sensitizes GC cells to CDDP. (a) The number of colonies formed by BGC823CDDP and SGC7901CDDP cells was reduced after miR-99a-5p knockdown. (b) DNA synthesis in BGC823CDDP and SGC7901CDDP cells was repressed by miR-99a-5p inhibition. (c) Apoptosis of CDDP-resistant GC cells transfected with miR-NC or miR-99a-5p inhibitor was detected by flow cytometric analysis. (d) The effect of miR-99a-5p on the apoptosis of BGC823CDDP and SGC7901CDDP cells was examined by western blotting. CDDP treatment: 12 μM for 48 h in BGC823CDDP cells and 6 μM for 48 h in SGC7901CDDP cells. (*p < 0.05, **p < 0.01. Data are expressed as the means ± SDs). [file 13046_2020_1758_MOESM5_ESM.pdf]
